# Supplementary material for: Optogenetic polymerization and assembly of electrically functional polymers for modulation of single-neuron excitability
Source: Sci Adv. 2022 Dec 7;8(49):eade1136. doi: 10.1126/sciadv.ade1136 (PMC9728971; doi:10.1126/sciadv.ade1136)
Supplement: 20221207-1 [file sciadv.ade1136.v1.pdf]

MATERIALS SCIENCE

# Optogenetic polymerization and assembly of electrically functional polymers for modulation of single-neuron excitability

Copyright © 2022 The Authors, some rights reserved; exclusive licensee American Association for the Advancement of Science. No claim to original U.S. Government Works. Distributed under a Creative Commons Attribution License 4.0 (CC BY-NC).

**Chanan D. Sessler[1,2]†, Yiming Zhou[2,3]†, Wenbo Wang[4]†, Nolan D. Hartley[2,3], Zhanyan Fu[2,3], David Graykowski[2,3], Morgan Sheng[2,3,5], Xiao Wang[1,2,3]*, Jia Liu[4]***

**Ionic conductivity and membrane capacitance are two foundational parameters that govern neuron excitability. Conventional optogenetics has emerged as a powerful tool to temporarily manipulate membrane ionic conductivity in intact biological systems. However, no analogous method exists for precisely manipulating cell membrane capacitance to enable long-lasting modulation of neuronal excitability. Genetically targetable chemical assembly of conductive and insulating polymers can modulate cell membrane capacitance, but further development of this technique has been hindered by poor spatiotemporal control of the polymer deposition and cytotoxicity from the widely diffused peroxide. We address these issues by harnessing genetically targetable photosensitizer proteins to assemble electrically functional polymers in neurons with precise spatiotemporal control. Using whole-cell patch-clamp recordings, we demonstrate that this optogenetic polymerization can achieve stepwise modulation of both neuron membrane capacitance and intrinsic excitability. Furthermore, cytotoxicity can be limited by controlling light exposure, demonstrating a promising new method for precisely modulating cell excitability.**

## INTRODUCTION

The intrinsic excitability of neurons is governed by two membrane properties: membrane conductivity and capacitance (*1*, *2*). Electrical, optogenetic, and pharmacological manipulations can transiently change membrane properties through manipulations of ion channels (*3*, *4*). In particular, conventional optogenetic stimulation harvests optical-driven ion channels [e.g., channelrhodopsin-2 (*5*) and halorhodopsin (*6*)], enabling millisecond time scale, genetically targeted, all-optical excitation and inhibition of living neurons (*7*–*10*). In contrast to transiently switching ion channels, naturally occurring neuron development and myelination processes suggest that modulating membrane capacitance (*11*–*13*) is another effective way of manipulating neuron intrinsic excitability during brain development, learning, and aging (*14*–*18*). Increasing/decreasing the membrane capacitance can decrease/increase the cellular excitability (*19*–*21*) and the velocity of action potential propagation (*22*–*25*).

Recent advances in materials science and nanotechnology have shown that the incorporation of miniaturized electrically functional materials and components (*26*–*32*) onto cellular membranes can modulate the membrane capacitance, changing the intrinsic cellular activities in vitro, which can potentially alter the neuron excitability in a long-term manner. However, these techniques do not enable genetically targeted specificity in neuronal circuits, partly due to the difficulty of incorporating prefabricated nanomaterials into biological systems in a cell type– or subcellular-specific manner. In vivo synthesis of functional nanomaterials has recently emerged as a promising alternative strategy for the integration of nanomaterials with living systems, often providing greater control over the location and integration of materials at the cellular level (*32*). Hence, we recently (*33*) modified an engineered peroxidase, Apex2, (*34*) to be expressed in genetically specified neurons in brain tissues. The peroxidase can catalyze the oxidative polymerization of small-molecule precursors into electrically functional (conductive or insulating) polymers at the plasma membrane in the presence of $H_2O_2$. Whole-cell patch-clamp showed that the in situ–synthesized conductive/insulating polymers increased/decreased the membrane capacitance and reduced/elevated the excitability of polymer-coated neurons, respectively. This method shows promise to change the excitability of specific types of neurons in intact neural circuits. However, this peroxidase/$H_2O_2$-driven polymerization has the following limitations: (i) Diffusion of $H_2O_2$ introduces acute toxicity to the neural systems, and (ii) the $H_2O_2$-triggered polymerization cannot control the location and extent of in situ polymerization in neurons. These limitations prevent the further application of this technique to living cell membrane modulation with cellular and subcellular spatiotemporal resolution.

Here, we address these issues by developing an optically controlled, genetically targeted (optogenetic) polymerization and assembly of conductive and/or insulating polymers on the neuronal plasma membrane, which, akin to conventional optogenetic stimulations, not only precisely modulates the membrane capacitance in a light-controlled and stepwise manner but also achieves cell type–specific control over neuron excitability. To enable this optogenetic polymerization of electrically functional synthetic polymers, we introduced genetically targetable photosensitizer proteins to photopolymerized polyaniline (PANI) and poly(3,3′-diaminobenzidine) (PDAB) as conductive and insulating polymers throughout the cell, respectively. We used whole-cell patch-clamp to characterize the electrophysiological properties of the same neurons before

[1]Department of Chemistry, Massachusetts Institute of Technology, Cambridge, MA, USA. [2]Broad Institute of MIT and Harvard, Cambridge, MA, USA. [3]Stanley Center for Psychiatric Research, Broad Institute of MIT and Harvard, Cambridge, MA, USA. [4]Harvard John A. Paulson School of Engineering and Applied Sciences, Harvard University, Cambridge, MA, USA. [5]Department of Brain and Cognitive Sciences, Massachusetts Institute of Technology, Cambridge, MA, USA.
*Corresponding author. Email: jia_liu@seas.harvard.edu (J.L.); xwangx@mit.edu (X.W.)
†These authors contributed equally to this work.

and after the optogenetic polymerization, showing that the in situ–synthesized conductive or insulating materials can increase or decrease the neuronal membrane capacitance and decrease or increase the intrinsic cellular excitability, respectively. Last, we showed that optogenetic polymerization can precisely control the location and density of polymers in cells by controlling the light intensity and exposure, thus enabling an iterative, stepwise increase/decrease of membrane capacitance.

## RESULTS

### Optogenetic polymerization of PDAB and PANI in living cells

We selected the photosensitizing protein mini Singlet Oxygen Generator (miniSOG) (*35*) to enable optical control of PDAB and PANI assembly on or within the cell membrane (Fig. 1A). Compared to other genetically targetable photosensitizers such as KillerRed and its derivatives (*36*, *37*), miniSOG produces more singlet oxygen relative to other reactive oxygen species (ROS) (*35*, *38*), resulting in its ability to photopolymerize DAB with nanometer-level spatial resolution (*35*). In addition, we hypothesize that this singlet oxygen generation could also synthesize the conductive polymer PANI inside cells (fig. S1). Several studies have reported the successful synthesis of PANI from a mixture of aniline and the aniline dimer *N*-phenyl-*p*-phenylenediamine (PPD) photosensitized by the singlet oxygen generator (*39*) $Ru(bpy)_3^{2+}$ in aqueous solution (*40*, *41*), suggesting the overall feasibility of photosensitized PANI polymerization. Although the participation of singlet oxygen in the reaction was not investigated, PPD, unlike monomeric aniline (*42*, *43*), has been shown to be an effective singlet oxygen quencher (*44*), and related *p*-phenylenediamine derivatives have been shown to react with singlet oxygen via a charge transfer in aqueous solution, generating the aminium radical cations (*44*) necessary for aniline polymerization (*45*, *46*).

Furthermore, the short lifetime of singlet oxygen in the aqueous solution implies that only a small (submicrometer scale) (*47*) region of the cell near the expressed miniSOG is exposed to the oxidant (*38*), suggesting that we can spatially confine the toxic effects of miniSOG. In contrast to previous chromophore-assisted light inactivation (CALI) studies in which miniSOG protein fusions have been used to perturb the function of specific protein targets under high light exposure (*48*, *49*), we hypothesized that carefully controlling light exposure and sparsely expressing miniSOG (Fig. 1A and fig. S2) can avoid both diffusive cytotoxicity and the specific inactivation of membrane proteins commonly targeted for CALI. In addition, quenching of singlet oxygen by the introduced DAB (*50*) or PPD (*44*) could also potentially limit the singlet oxygen exposure.

To assess the ability of miniSOG to polymerize DAB and PPD in living cells, we first expressed miniSOG, joined with dTomato by a T2A ribosomal skip sequence, in the cytosol of human embryonic kidney (HEK) 293T cells under the control of the CAG promoter. Irradiating fixed cells expressing this miniSOG construct (termed as miniSOG$^+$ cells) with a standard green fluorescent protein (GFP) filter set in the presence of 1 mM DAB produced the expected dark brown precipitate in transmitted light (TL) images where miniSOG fluorescence was observed (Fig. 1, B and D). As expected, darkening due to PDAB assembly correlated well with miniSOG expression, with nontransfected cells showing essentially no polymerization (fig. S3A). Similarly, when fixed miniSOG$^+$ cells were irradiated in a mixture of aniline and PPD, a lighter purple-gray precipitate indicative of PANI formed in only the irradiated cells (Fig. 1, C and D). Compared with PDAB, PANI polymerization also occurred primarily in transfected cells, but some nonspecific polymerization was observed in nontransfected cells, potentially resulting from nonsensitized photo-oxidation of PPD. An increased monomer concentration (1 mM each of aniline and PPD) was required to achieve a similar level of polymerization as characterized by the darkening of the reacted cells (fig. S3B). Similar results were obtained in living cells for both polymer types, albeit with higher nonspecific background polymerization in the case of PANI (fig. S3, C to G). Notably, a clear boundary between polymerized and nonpolymerized regions of cells was visible for PDAB (fig. S3E), although a small amount of polymerization is observed outside the region exposed to the highest light intensity as a result of the noncoherent light source used. This demonstrates that a subcellular-level spatially specific polymerization is achievable by optogenetic polymerization in living cells.

We next used ultraviolet-visible (UV/vis) spectroscopy to characterize the in situ optogenetically polymerized PANI and PDAB. The polymerized PANI displayed features consistent with chemically synthesized, undoped PANI (*51*), while PDAB exhibited a broad absorbance in the visible range consistent with literature reports (fig. S3H) (*33*). Notably, the PANI deposited on cells exhibited a red shift consistent with chemically synthesized PANI upon acid-doping treatment with hydrogen chloride (fig. S3H), suggesting similar extended, conjugated structures for the miniSOG-catalyzed PANI formed on the cells (*33*, *51*). Consistent with our proposed mechanism, miniSOG-catalyzed PANI polymerization appeared to be primarily dependent on singlet oxygen. Specifically, deoxygenated monomer solutions formed little to no detectable PANI precipitate (fig. S4), as did photosensitizers primarily producing ROS other than singlet oxygen (fig. S5), demonstrating the importance of a singlet oxygen-producing photosensitizer for in situ PANI polymerization.

Because TL darkening and electron microscopy both require a high amount of PDAB deposition for visualization, we devised a more sensitive and direct method for assessing the location of the deposited PDAB by fluorescently labeling the deposited polymer. This could not be achieved by directly tethering the DAB monomer to fluorophores, mainly due to the instability of most fluorophores toward singlet oxygen. Here, we instead tethered the DAB monomer to biotin via a polyethylene glycol linker (figs. S6 and S7, A and B). When miniSOG$^+$ HEK293T cells were illuminated as above in the presence of 1 mM biotin-DAB conjugate, followed by staining with a streptavidin–Alexa Fluor 647 (AF647) conjugate, the AF647 signal was primarily observed in the darkened, light-exposed cells, and a clear boundary of illumination was present (fig. S7C). Unlike TL imaging, streptavidin visualization of biotin-PDAB allowed clear three-dimensional reconstruction of the PDAB location with confocal microscopy, demonstrating a cytosolic distribution of the deposited PDAB, albeit with greater PDAB deposition near or in the cell membrane (fig.S7D). Although miniSOG was not targeted to the cell membrane, it is likely that the PDAB and PANI tended to accumulate within the hydrophobic core of the lipid bilayer due to their high lipophilicity.

We next constructed and packaged adeno-associated virus (AAV) containing the miniSOG construct under the control of

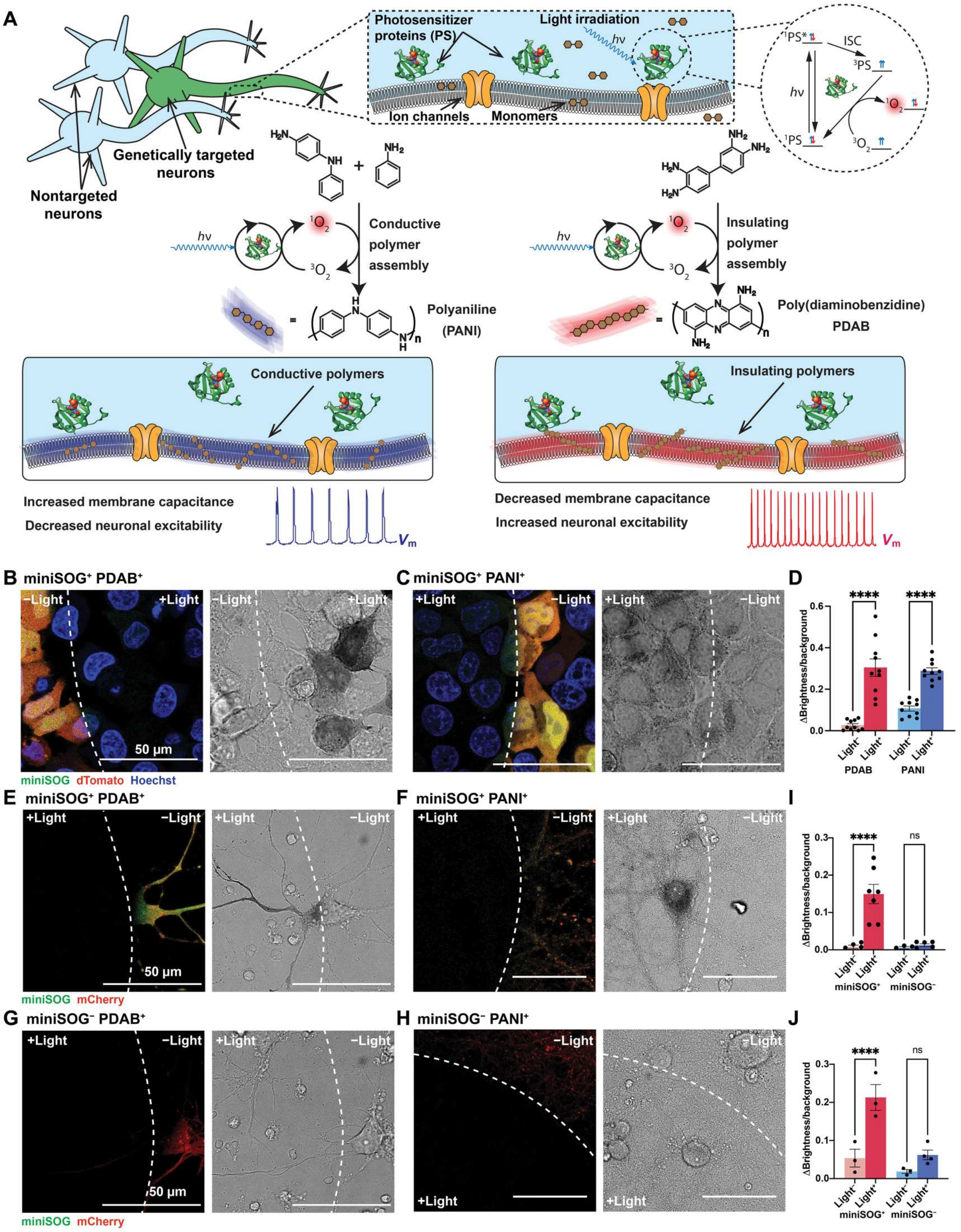


**Fig. 1. Optogenetic polymerization and assembly of electrically functional materials in cells.** (**A**) Photosensitizer proteins (PS) are expressed in the cytosol of the genetically specified neuron types. Upon light irradiation (*hv*) and introduction of polymer monomers, photosensitizer proteins locally generate singlet oxygen ($^1O_2$)after instersystem crossing (ISC), which polymerizes and assembles conductive or insulating polymers in the cell and on the cell membrane, modulating the membrane capacitance and excitability ($V_m$, membrane potential). (**B** and **C**) Merged confocal fluorescence (left) and transmitted light (TL; right) images of fixed human embryonic kidney (HEK) 293T cells coexpressing dTomato (red) and miniSOG (green) after 7 min of irradiation in the presence of 1 mM DAB [labeled as poly(3,3′-diaminobenzidine) (PDAB$^+$)] (B) and 1 mM aniline and 1 mM *N*-phenyl-*p*-phenylenediamine (PPD) [labeled as photopolymerized polyaniline (PANI$^+$)] (C). White dashed lines represent the boundary of irradiation formed by the epifluorescence light source; Hoechst 33342 nuclear stain is shown in blue; all scale bars are 50 μm. (**D**) PDAB and PANI depositions were quantified by measuring the pixel brightness of cells in the TL images and normalizing it to the average background of the cell-free parts of the image. Values represent means ± SEM; unpaired, two-tailed *t* tests. ****$P < 0.0001$. (**E** and **F**) Merged confocal fluorescence and TL images of fixed rat cortical neurons expressing miniSOG (miniSOG$^+$ neurons) after 7 min of irradiation in the presence of 1 mM DAB (E) and 1 mM aniline and 1 mM PPD (F). (**G** and **H**) Corresponding images of neurons expressing only mCherry (miniSOG$^-$ neurons). (**I** and **J**) Insulating (I) and conductive (J) polymer deposition quantified as described in (D). ns, not significant.

the human synapsin promoter for infection of in vitro–cultured rat cortical neurons. We infected neurons with $10^{10}$ viral genomes (vg) of AAV (AAVdj) packaged with the miniSOG construct or AAVdj packaged with mCherry as controls (multiplicity of infection = $1 \times 10^5$) at 14 days in vitro (DIV), followed by 3 to 5 days of expression after infection. As in HEK293T cells, irradiation of fixed neurons expressing the intracellular miniSOG in the presence of 1 mM DAB resulted in a dark brown precipitate only in the regions exposed to light by the objective (Fig. 1E). When fixed neurons were instead exposed to light in the presence of the aniline and PPD mixture, the corresponding purple-gray precipitate was observed (Fig. 1F). Neurons expressing only mCherry (hereafter miniSOG$^-$) formed little to no precipitate when irradiated in the presence of either monomer solution (Fig. 1, G to J).

## Biocompatibility of optogenetic polymerization

We proceeded to examine the effects of miniSOG-catalyzed polymerization of conductive and insulating polymers on neuron electrophysiology. First, we tested whether miniSOG-catalyzed polymerization can be achieved effectively and specifically in miniSOG$^+$ neurons for electrophysiological recordings. Specifically, we infected neurons as described above, but to avoid the potential toxicity of miniSOG (*52*, *53*) by achieving a weak expression level, we restricted the infection period to 3 to 5 days. At 14 DIV, the primary neurons showed a pyramidal shape and stellate shape (*54*), indicating an in vitro maturation (Fig. 2, A and B). We then replaced the medium with Tyrode's solution containing monomers (1 mM DAB or a mixture of 0.5 mM each of aniline and PPD) and irradiated the neurons with a 475-nm blue light for 5 to 7 min to polymerize PDAB or 9 to 15 min to polymerize PANI. Notable precipitates of either DAB or PANI can be observed by the obvious darkening of membranes of miniSOG$^+$ neurons but not miniSOG$^-$ control (Fig. 2, A and B).

To assess whether the miniSOG-catalyzed polymerization of PANI and PDAB is biocompatible with neurons, we performed cell viability tests and electrophysiology characterization on neurons before and after polymerization. The same polymerization conditions for PDAB and PANI were applied. Specifically, for the cell viability test, calcein acetoxymethyl ester (calcein AM) and NucRed probes were added into the medium and incubated for 30 min to stain live and dead neurons, respectively (Fig. 2C). No statistically significant difference was detected between any group, regardless of the presence of miniSOG or each monomer (Fig. 2D and fig. S8), indicating that the polymerization reaction as performed here (irradiation for 7 min with approximately 5 mW/mm$^2$) has no acute cytotoxic effects on the viability of neurons.

Then, we characterized the electrophysiology of the cultured primary rat cortical neurons before and after the miniSOG-catalyzed polymerization by whole-cell patch-clamp measurement. Notably, compared with a previous Apex2-catalyzed polymerization (*33*), the miniSOG-catalyzed polymerization allowed us to directly measure the same neurons before and after polymerization. During electrophysiological measurements, each neuron was maintained in whole-cell patch mode throughout the entire recording procedure (Fig. 3A). It was reported that long-term irradiation of miniSOG itself can potentially influence neuronal electrophysiological activity (*48*, *55*). We also observed that, when the miniSOG expression was high or irradiation duration was long, neurons exhibited hyperexcitability after irradiation in the absence of monomers (fig. S9). Therefore, to avoid the side effects from the irradiation of miniSOG, we first tested conditions that would least affect neuronal health and proper functioning before comparing the effects of polymerization on miniSOG$^+$ and miniSOG$^-$ neurons. In this measurement, miniSOG$^+$ neurons were recorded in the absence of monomers with differing irradiation time (fig. S10A). We manually selected pyramidal-like neurons with low expression of miniSOG-mCherry by fluorescence intensity [corrected total mCherry fluorescence of approximately $<1.3 \times 10^4$ arbitrary units under green light (~7 mW/mm$^2$) as in fig. S9C] and found that up to 7 min of blue light irradiation (5 mW/mm$^2$) has little to no effect on the electrophysiological properties of miniSOG$^+$ neurons (fig. S10, B to D). Specifically, comparing the same neurons before and after polymerization, we did not detect significant changes in neuronal membrane capacitance (74.59 ± 5.88 versus 74.00 ± 6.50 pF, $P > 0.05$; before versus after irradiation, means ± SEM, reported throughout this paper, unless otherwise stated, $n = 7$), resistance (383.57 ± 63.91 versus 366.14 ± 85.75 megohms, $n = 7$, $P > 0.05$), and rheobase (97.14 ± 12.67 versus 128.57 ± 25.77 pA, $n = 7$, $P > 0.05$) (fig. S10B). In addition, the action potential spike number at rheobase, rheobase + 40 pA, and rheobase + 80 pA was not significantly changed (2.5 ± 0.43 versus 3.7 ± 0.99, 8 ± 0.68 versus 8.3 ± 1.2, and 11.2 ± 0.98 versus 11.5 ± 1.3 pA, $n = 7$, $P > 0.05$), suggesting no alterations in cellular excitability (fig. S10C). Furthermore, the half-width duration and the amplitude of current-elicited action potentials were not significantly altered before and after irradiation (half-width duration: 1.49 ± 0.11 versus 2.19 ± 0.37 ms; amplitude: 112.25 ± 4.71 versus 110.32 ± 4.84 mV, $n = 7$, $P > 0.05$) (fig. S10, C and D). However, the latency of phasic stimulation-induced action potentials was significantly decreased (5.87 ± 0.60 versus 4.99 ± 0.72 ms, $n = 7$, $P = 0.038$), suggesting that there could be some unanticipated effects resulting from miniSOG irradiation. Despite some effects on latency, these results demonstrate that 7 min of 475-nm light irradiation (5 mW/mm$^2$) of neurons with controlled miniSOG expression will not generally affect neuron electrophysiological behaviors as measured by the whole-cell patch-clamp. Although the PDAB precipitate formed under these weak expression and irradiation conditions is challenging to visualize by TL imaging, streptavidin staining of miniSOG$^+$ neurons polymerized with biotin-DAB under these conditions revealed robust PDAB deposition (fig. S7, E to I). Similarly, when these irradiation conditions were applied to miniSOG$^-$ neurons, no significant changes in any of the above electrophysiological properties were observed (fig. S11). Therefore, we further applied this AAVdj infection and optical irradiation condition to the following optogenetic polymerization experiments (Fig. 3A).

## Modulation of neuron excitability by optogenetic polymerization

Next, we examined whether the miniSOG-catalyzed polymerization of PDAB and PANI could alter the electrophysiological behaviors of neurons. Specifically, the inclusion of conductive or insulating polymers on and within the cell membrane is likely to increase or decrease the capacitance of the membrane, respectively, which can be modeled as $C = \epsilon\frac{A}{d}$, in which $C$, $\epsilon$, $A$, and $d$ are the membrane capacitance, permittivity, surface area, and distance of separation, respectively. In the case of conductive polymers (PANI), the large permittivity of these materials (ca. $10^4$) (*56*) would be expected to

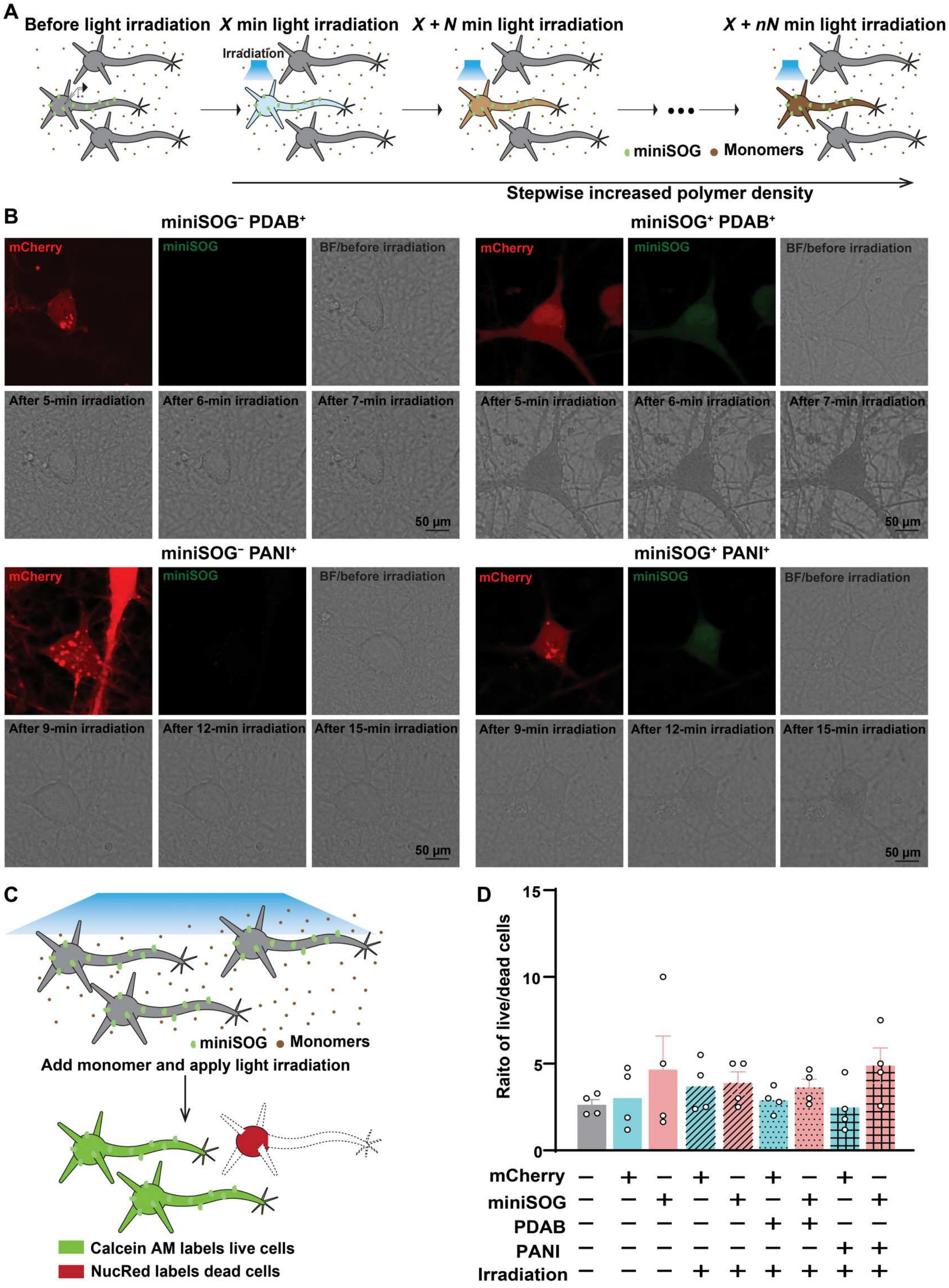


**Fig. 2. Optogenetic polymerization and assembly of electrically functional polymers in living neurons.** (**A**) Schematics of iterative optogenetic polymerization in living neurons, where *X* represents the initial irradiation time, *N* represents the added irradiation time in subsequent irradiation rounds, and *X* + *nN* represents the total irradiation time after *n* rounds of irradiation. (**B**) Representative confocal images showing primary miniSOG$^{+}$ and miniSOG$^{-}$ neurons before and after PDAB (top) or PANI (bottom) optogenetic polymerization. The optogenetic polymerization was performed by immersing the neurons in Tyrode's solution containing 1 mM DAB or 0.5 mM aniline-PPD mixture at 1:1 molar ratio and being irradiated with blue light for 5 to 7 min (PDAB reaction) or 9 to 15 min (PANI reaction). (**C**) Schematics illustrate the viability test of cells after miniSOG-catalyzed polymerization under different conditions by live/dead ratio assay. (**D**) Statistical results of live/dead ratio calculated from the nine groups. Four replicates per group were performed. Values represent the ratio of live/dead cells; means ± SEM. Unpaired two-tailed *t* tests. all $P > 0.05$. Light intensities used for miniSOG-catalyzed polymerization are approximately 5 mW/mm$^2$.

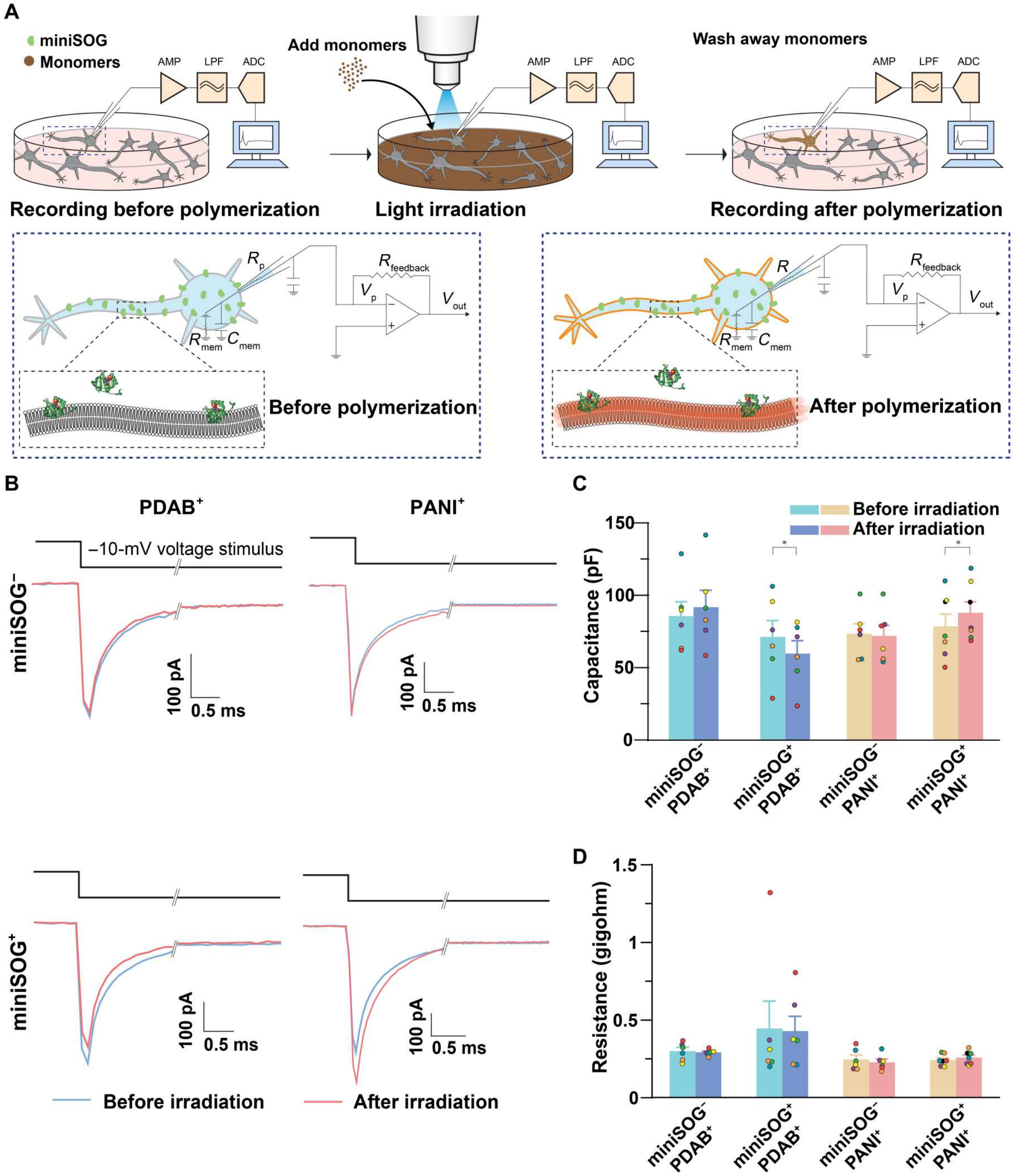


**Fig. 3. Optically controlled and genetically targeted modulation of single-neuron excitability.** (**A**) Schematics of electrophysiological characterization on the cultured neurons. Top: Whole-cell recordings were performed in the Tyrode's solution in the absence of monomers (step 1), and then neurons were perfused with Tyrode's solution containing 1 mM DAB or 0.5 mM each aniline and PPD. Neurons were then irradiated with blue light (step 2). After irradiation, monomer solutions were washed away by normal Tyrode's solution. During the entire process, neurons were maintained under whole-cell clamp mode for recording (step 3). The setup for whole-cell recording includes an amplifier (AMP), a low-pass filter (LPF), and an analog-to-digital converter (ADC). Bottom: Schematics of the recorded neuron showing the membrane properties measured by whole-cell patch-clamp before and after optogenetic polymerization. $V_p$ is the pipette voltage, $R_p$ is the pipette resistance, $C_p$ is the pipette capacitance, $R_{feedback}$ is the resistance of a feedback resistor, $R_{mem}$ is the membrane resistance, $C_{mem}$ is the membrane capacitance, and $V_{out}$ is the output voltage. (**B**) Representative current responses evoked by a 10-mV hyperpolarization step in voltage clamp before and after PDAB (top) or PANI polymerization (bottom). Cells were held at −70 mV and then hyperpolarized to −80 mV for 500 ms followed by −70 mV holding. The charge and discharge current waveforms were analyzed to extract the capacitance and resistance. (**C** and **D**) Comparison of membrane capacitance (C) and resistance (D) before and after PDAB or PANI polymerization ($n$ = 6 neurons for miniSOG$^-$/PDAB, miniSOG$^+$/PDAB, and miniSOG$^-$/PANI groups and $n$ = 7 neurons for miniSOG$^+$/PANI group). Bar graphs represent means ± SEM; dots with the same color within each group indicate the same neuron. Paired two-tailed $t$ test. *$P < 0.05$; $t$ tests with nonsignificant $P$ values are omitted for clarity.

increase the permittivity of the membrane upon assembly within the membrane, increasing the capacitance accordingly. In contrast, the incorporation of insulating polymers (PDAB), which have a much lower permittivity, would lead to a decrease in capacitance upon incorporation within the membrane. Note that PDAB is also known to accumulate outside of membranes (*35*), which would primarily modulate *d*, but this would also lead to decreases in capacitance in accordance with increasing membrane thickness. According to the Hodgkin-Huxley model, membrane capacitance can be regarded as an inversely proportional scale factor that influences the sensitivity of the changes in membrane potential in response to external stimuli (*2*, *21*). These increases or decreases in membrane capacitance should then be reflected as decreases or increases in neuron excitability, respectively (*19*).

We characterized these optogenetic polymerization and assembly-induced changes in membrane properties using whole-cell patch-clamp recordings. The above light irradiation conditions were applied to neurons recorded in whole-cell patch mode in the presence of either the PDAB or PANI precursor solutions, permitting electrophysiological characterization of the same neuron before and after polymer assembly. To avoid any side effects on the membrane properties from remaining monomers after optogenetic polymerization, cell electrophysiology was measured after removing the monomer solution. Directly following the light irradiation, we observed pronounced changes in the square voltage step-induced current waveform from miniSOG$^+$ neurons, but no change from miniSOG$^-$ neurons (Fig. 3B). Notably, irradiated miniSOG$^+$/PDAB neurons had a significant decrease in membrane capacitance (71.33 ± 11.41 versus 59.93 ± 8.91 pF, $n = 6$, $P = 0.027$), whereas irradiated miniSOG$^+$/PANI neurons had a significant increase in membrane capacitance (78.82 ± 8.33 versus 88.11 ± 7.56 pF, $n = 7$, $P = 0.021$). No statistically significant changes of membrane capacitance were detected from irradiated miniSOG$^-$/PDAB (85.42 ± 9.97 versus 91.57 ± 11.53 pF, $n = 6$, $P > 0.05$) and miniSOG$^-$/PANI neurons (73.62 ± 6.90 versus 72.15 ± 7.29 pF, $n = 6$, $P > 0.05$) (Fig. 3, B and C). Meanwhile, we did not notice a significant change of the membrane resistance from all neurons after optical irradiation, indicating that light exposure or the resulting polymerization in these conditions does not alter whole-cell conductance (Fig. 3, B and D).

We then examined whether the optogenetic polymerization can increase or decrease the intrinsic neuronal excitability in accordance with the changes in membrane capacitance. As expected, the irradiated miniSOG$^+$/PDAB neurons exhibited increased current injection–evoked action potential firing to depolarizing stimuli (the spike number increased from 2.33 ± 0.61 to 4.83 ± 1.19 at rheobase, $P = 0.037$; from 6.33 ± 0.80 to 8.50 ± 0.92 at rheobase + 40 pA, $P = 0.010$; and from 8.17 ± 1.40 to 11.17 ± 0.75 at rheobase + 80 pA, $P = 0.023$, $n = 6$) (Fig. 4, A to C), whereas the irradiated miniSOG$^+$/PANI neurons exhibited decreased action potential firing to depolarizing stimuli (the spike number decreased from 1.71 ± 0.36 to 1.57 ± 0.92 at rheobase, $P = 0.90$; from 5.57 ± 0.95 to 1.57 ± 0.37 at rheobase + 40 pA, $P = 0.0064$; and from 8.00 ± 0.72 to 1.86 ± 0.55 at rheobase + 80 pA, $P = 0.0013$, $n = 7$) (Fig. 4, B and C). In contrast, the irradiated miniSOG$^-$/PDAB and miniSOG$^-$/PANI did not result in a significant change in the current injection–evoked spike numbers. Notably, we did not observe changes in the average rheobase level (current spiking threshold) from all samples (fig. S12B), consistent with the lack of an effect on membrane resistance. Collectively, these results demonstrate that optogenetic PDAB and PANI assembly can elicit both increases and decreases in the intrinsic excitability of neurons.

We also systematically characterized the stimulation-elicited intracellular action potentials of neurons before and after optogenetic polymerization by applying brief phasic current stimulation. We did not detect significant changes in the amplitude and kinetics of phasic current–evoked responses after PDAB polymerization (fig. S12, C to F). However, we observed a significant increase of the spike half-width duration (from 1.61 ± 0.09 to 2.55 ± 0.24 ms, $n = 7$, $P = 0.0087$) in miniSOG$^+$/PANI group, while the miniSOG$^-$/PANI group did not show a statistically significant change (1.47 ± 0.14 versus 1.49 ± 0.19, $n = 6$, $P > 0.05$) (fig. S12F). The increase of action potential spike half-width suggests the decrease of neuronal excitability as a result of decreased depolarization and repolarization slopes (*57*).

Next, we sought to characterize the long-term effects of PDAB and PANI polymerization on neuron viability and electrophysiology at both 1 and 3 days after polymerization. Because the neurons cannot be held under whole-cell patch for this duration, we instead compared the electrophysiology between miniSOG$^-$/monomer$^-$, miniSOG$^+$/monomer$^-$, miniSOG$^+$/PDAB, and miniSOG$^+$/PANI neurons at the population level at each time point after polymerization (fig. S13A and S14A). Viability of miniSOG$^+$/PDAB and miniSOG$^+$/PANI neurons was not significantly affected at either 1 or 3 days after polymerization, although the viability of miniSOG$^+$/monomer$^-$ neurons did decrease significantly after 3 days likely due to the absence of singlet oxygen quenching by the monomers (figs. S13B and S14B). Patch-clamp recordings revealed significantly higher capacitance in the miniSOG$^+$/PANI neurons compared to miniSOG$^+$/PDAB neurons at 1 day after polymerization (60.11 ± 8.20 versus 39.75 ± 3.68 pF, $n = 6$, $P = 0.047$, unpaired two tailed *t* test) without significant changes in resistance or rheobase (fig. S13, C to E). At 3 days after polymerization, the overall trend in capacitance was retained, albeit not statistically significant (55.79 ± 8.14 versus 43.66 ± 5.53 pF, $n = 6$, $P = 0.246$, unpaired two tailed *t* test) with no clear differences in resistance or rheobase (fig. S14, C to E).

In terms of excitability, the spike number when held at rheobase + 80 pA was significantly lower in the miniSOG$^+$/PANI neurons compared to miniSOG$^+$/PDAB neurons at both 1 day after polymerization (3.83 ± 1.52 versus 9.00 ± 0.82, $n = 6$, $P = 0.013$, unpaired two-tailed *t* test) and 3 days after polymerization (2.667 ± 0.61 versus 6.00 ± 1.03, $n = 6$, $P = 0.020$, unpaired two-tailed *t* test), while at rheobase and rheobase + 40 pA, the trend was the same but not statistically significant at either time point (figs. S13, F to H; and S14, F to H). As expected, no statistically significant differences in spike latency, half-width duration, or amplitude were observed between any groups at either time point (figs. S13, I to K; and S14, I to K). These results indicate that optogenetic PDAB and PANI polymerization are capable of capacitance-induced changes in neuron excitability lasting days after polymerization, without affecting neuron viability. In addition, the long-term stability of these electrophysiological changes suggests that the polymers tend to accumulate within the membrane itself, as opposed to on the surface of the membrane, where gradual diffusion throughout the cytosol (*58*) would be expected to reverse the effects of optogenetic polymerization over time.

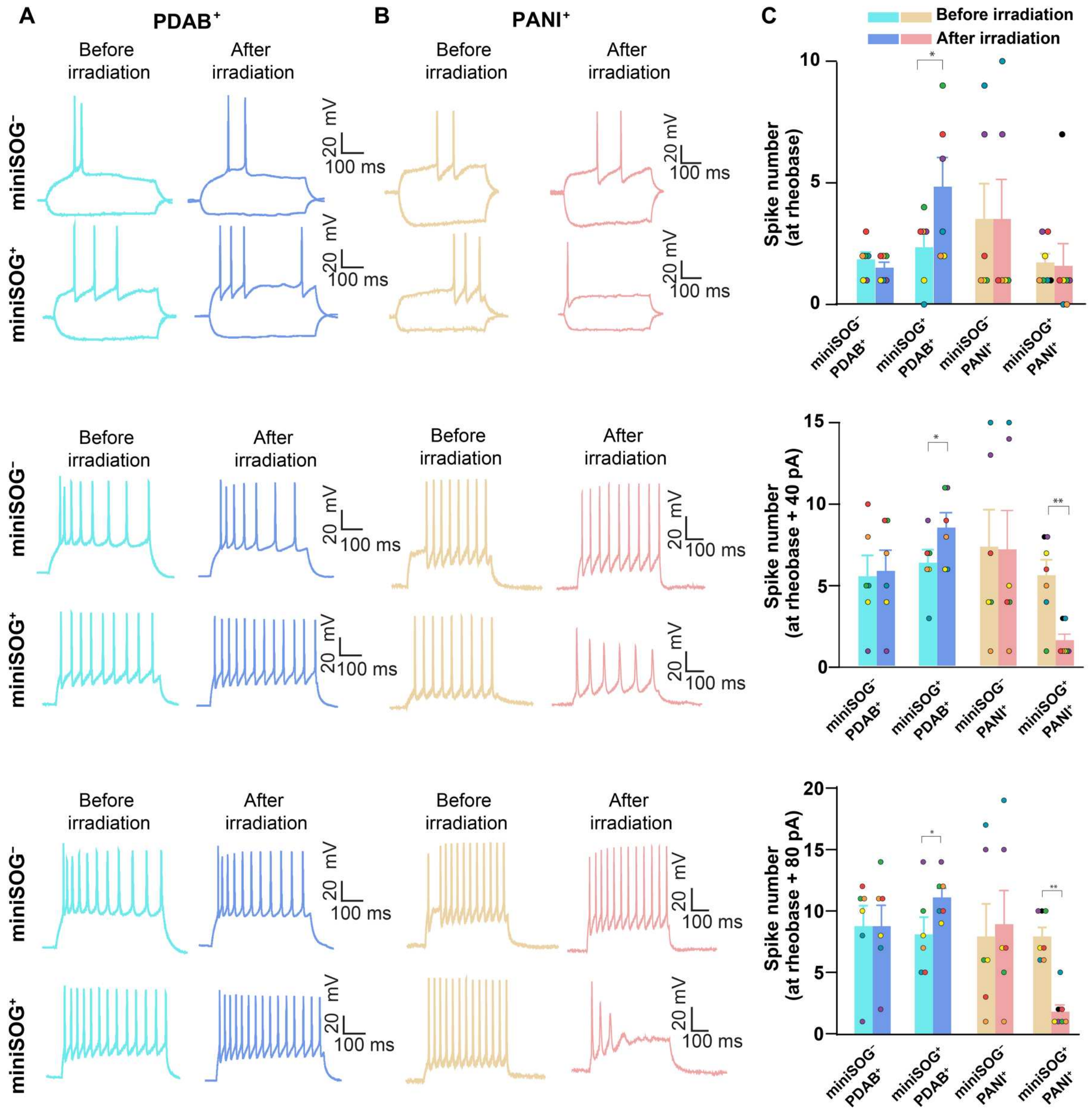


**Fig. 4. Current injection–evoked spikes characterization before and after optogenetic polymerization of functional polymers in living neurons.** Same polymerization procedures were applied on cultured primary miniSOG$^+$- and miniSOG$^-$-cultured neurons as those in Fig. 2. (**A**) Representative traces evoked by stepwise tonic current injection (20 pA per step from −100 to 280 pA) before and after PDAB polymerization reaction. From top to bottom, action potentials were evoked at rheobase, rheobase + 40 pA, and rheobase + 80 pA in the current clamp mode, respectively. (**B**) Representative traces evoked by stepwise tonic current injection (20 pA per step from −100 to 280 pA) before and after PANI polymerization. The traces were recorded under current clamp mode. Cells were held at −70 to −75 mV potential by injecting current. Stepwise tonic current (20 pA per step from −100 to 280 pA) was injected into cells to elicit action potentials and to determine rheobases. From top to bottom, action potentials were evoked at rheobase, rheobase + 40 pA, or rheobase + 80 pA, respectively. (**C**) Comparison of spike numbers before and after PDAB or PANI polymerization at rheobase, rheobase + 40 pA, or rheobase + 80 pA in current clamp ($n = 6$ neurons in miniSOG$^-$/PDAB$^+$ group, $n = 6$ neurons in miniSOG$^+$/PDAB group, $n = 6$ neurons in miniSOG$^-$/PANI, and $n = 7$ neurons in miniSOG$^+$/PANI group). All individual cells were maintained in the whole-cell patch-clamp configuration across pre- and postreaction time points for direct comparison. Bar graphs represent means ± SEM; dots with the same color within each group indicate the same neuron. Paired two-tailed *t* test. $^*P < 0.05$ and $^{**}P < 0.01$; *t* tests with nonsignificant *P* values are omitted for clarity.

## Stepwise control of optogenetic polymerization and neuron excitability

Optogenetic polymerization can provide fine temporal control over the polymerization reaction, thus potentially allowing for stepwise fine-tuning of membrane properties by controlling the length of light exposure. To demonstrate that the optogenetic polymerization can be controlled in a predictable, stepwise manner, we measured the stepwise change of the membrane properties by continuously measuring the cultured primary neurons in the monomer solution during iterative optogenetic polymerization. After characterizing the initial membrane properties and electrophysiological behaviors of the neurons, we sequentially measured the membrane properties

and electrophysiological behaviors of the same neurons after 5, 6, and 7 min of irradiation (Fig. 5A). Note that, unlike the aforementioned single time point measurements, the stepwise changes were recorded in the presence of monomers due to the difficulty of washing and reperfusing the monomer with each measurement. We found that the capacitance of miniSOG$^+$ neurons was decreased in a stepwise manner during the PDAB polymerization (miniSOG$^+$/PDAB, before irradiation: 74.98 ± 9.08 pF; 5-min irradiation: 72.12 ± 9.10 pF, $P$ = 0.013 versus before irradiation; 6-min irradiation: 69.31 ± 9.03 pF, $P$ = 0.005 versus before irradiation; and 7-min irradiation: 67.35 ± 8.15 pF, $P$ = 0.009 versus before irradiation; $n$ = 6) and increased in a stepwise manner during the PANI polymerization (miniSOG$^+$/PANI, before irradiation: 87.21 ± 7.97 pF; 6-min irradiation: 91.80 ± 5.73 pF, $P$ = 0.22 versus before; 7-min irradiation: 94.04 ± 5.93 pF, $P$ = 0.067 versus before; and 8-min irradiation: 95.03 ± 5.97 pF, $P$ = 0.058 versus before; $n$ = 7) (Fig. 5, B and D). However, this trend is not statistically significant in the case of PANI likely due to the transient effects of the monomer solution itself on neuron electrophysiology. Similar to our previous results, the membrane resistances of irradiated miniSOG$^-$/PDAB and miniSOG$^-$/PANI neurons did not show significant changes over time (Fig. 5, C and E).

The spike numbers of irradiated miniSOG$^+$/PDAB neurons also increased significantly during the stepwise PDAB polymerization at the rheobase level (miniSOG$^+$/DAB, before irradiation: 2.33 ± 0.49; 5-min irradiation: 2.5 ± 0.43, $P$ = 0.77 versus before; 6-min irradiation: 3.33 ± 0.99, $P$ = 0.45 versus before; and 7-min irradiation: 4.00 ± 0.77, $P$ = 0.03 versus before; $n$ = 6), at rheobase level + 40 pA (miniSOG$^+$/DAB, before irradiation: 7.00 ± 1.07; 5-min irradiation: 7.67 ± 1.23, $P$ = 0.52 versus before; 6-min irradiation: 8.50 ± 1.46, $P$ = 0.39 versus before; and 7-min irradiation: 9.50 ± 0.99, $P$ = 0.042 versus before; $n$ = 6), and at rheobase level + 80 pA (miniSOG$^+$/DAB, before irradiation: 9.17 ± 1.82; 5-min irradiation: 10.33 ± 1.82, $P$ = 0.20 versus before; 6-min irradiation: 11.83 ± 1.58, $P$ = 0.20 versus before; and 7-min irradiation: 12.50 ± 1.18, $P$ = 0.045 versus before; $n$ = 6) (Fig. 5, F to H). Conversely, the spike numbers of irradiated miniSOG$^+$/PANI neurons showed a stepwise decrease during the PANI polymerization at rheobase level (miniSOG$^+$/PANI, before irradiation: 1.71 ± 0.42; 6-min irradiation: 1.29 ± 0.18, $P$ = 0.36 versus before; 7-min irradiation: 1.00 ± 0.22, $P$ = 0.14 versus before; and 8-min irradiation: 0.57 ± 0.20, $P$ = 0.030 versus before; $n$ = 7), at rheobase level + 40 pA (miniSOG$^+$/PANI, before irradiation: 4.29 ± 1.27; 6-min irradiation: 2.71 ± 1.06, $P$ = 0.025 versus before; 7-min irradiation: 2.29 ± 1.02, $P$ = 0.033 versus before; and 8-min irradiation: 2.00 ± 1.02, $P$ = 0.0068 versus before; $n$ = 7), and at rheobase level + 80 pA (miniSOG$^+$/PANI, before irradiation: 4.71 ± 1.30; 6-min irradiation: 3.29 ± 1.23, $P$ = 0.046 versus before; 7-min irradiation: 2.71 ± 1.25, $P$ = 0.068 versus before; and 8-min irradiation: 2.14 ± 0.86, $P$ = 0.035 versus before; $n$ = 7) (Fig. 5, I to K). Consistent with our previous results, the irradiated miniSOG$^-$/PDAB or miniSOG$^-$/PANI control neurons did not show significant changes for all measures (Fig. 5, F to K), and the membrane resistance or rheobase did not change significantly in miniSOG$^+$ and miniSOG$^-$ neurons during PDAB or PANI polymerization (Fig. 5, C and E, and fig. S15, B and F). In addition, the phasic current–induced response after stepwise PDAB polymerization showed no changes on the evoked action potential spike amplitude (fig. S15C). However, the latency decreased from 4.91 ± 0.61 ms (before irradiation) to 4.88 ± 0.93 ms ($P$ = 0.93 versus before), 4.45 ± 0.79 ms ($P$ = 0.15 versus before), and 3.98 ± 0.69 ms ($P$ = 0.007 versus before) after 5-, 6-, and 7-min irradiation ($n$ = 6) (fig. S15E), which suggests a higher likelihood to elicit action potentials due to increased neuronal excitability after PDAB polymerization. Meanwhile, the half-width duration increased from 1.42 ± 0.11 ms (before irradiation) to 1.50 ± 0.13 ms ($P$ = 0.086 versus before), 1.66 ± 0.15 ms ($P$ = 0.028 versus before), and 1.98 ± 0.28 ms ($P$ = 0.052 versus before) after 5-, 6-, and 7-min irradiation ($n$ = 6) (fig. S15D). We did not detect significant changes in the amplitude or kinetics of phasic current–evoked action potential after stepwise PANI polymerization on both miniSOG$^+$ and miniSOG$^-$ neurons (fig. S15, G to I). Together, these results demonstrate the ability to modulate membrane capacitance and excitability stepwise by iterative optogenetic polymerization of conductive and insulating polymers.

## DISCUSSION

Here, we introduce a method for modulating neural activity using optogenetic polymerization and assembly of electroactive polymers on specified cellular membranes. Compared with the previous neuromodulation method based on peroxidase catalysis, this optogenetic polymerization strategy provides spatiotemporal control over the polymer generation and assembly and reduces the broad toxicity of hydrogen peroxide. Furthermore, extending this photosensitized chemical assembly strategy to genetically targetable photosensitizers such as the Fluorogen Activating Protein - Targetable and Activatable Photosensitizers (FAP-TAPs) (*59*) with varying excitation wavelengths potentially allows for simultaneous, multicolor manipulation of different types of cells in intact neural circuits. Continued development of genetically targetable photosensitizers could enable polymer assembly with subcellular specificity and lower diffuse cytotoxicity. Engineering the structures of the monomers and oligomers can also further improve the biocompatibility and extend their functionalities, including potential degradability. We expect that these developments in biocompatible, in vivo polymer assembly will further allow this technique to be applied to in vivo manipulation of neuronal circuits and animal behaviors.

Notably, in contrast to the transient modulation of neuron activities achieved by manipulating ion channel conductivities, material-based manipulation of membrane capacitance (*1*, *11*–*13*) can modulate the neuronal electrophysiology in a long-term stable manner, at a time scale relevant to brain development, learning, and aging. Future development of photosensitized in situ biosynthesis of functional polymers in vivo could enable bidirectional neuromodulation to control brain activities and freely behaving animal behaviors such as precisely tuning the intrinsic excitation-inhibition balance within cortical microcircuits over longer time scales. Furthermore, it may ultimately provide electrotherapeutic stimulation options to ameliorate neurodegenerative and myelination degenerative diseases in a long-term stable manner through the restoration of cell excitability and action potential propagation. Last, the advances in biocompatible functional polymer synthesis presented here demonstrate the utility of in situ nanomaterial synthesis and assembly as an emerging synthetic biology technique for interfacing biological systems with synthetic materials. This can potentially provide far greater control over the integration of these materials at the cellular level than is typically afforded by conventional,

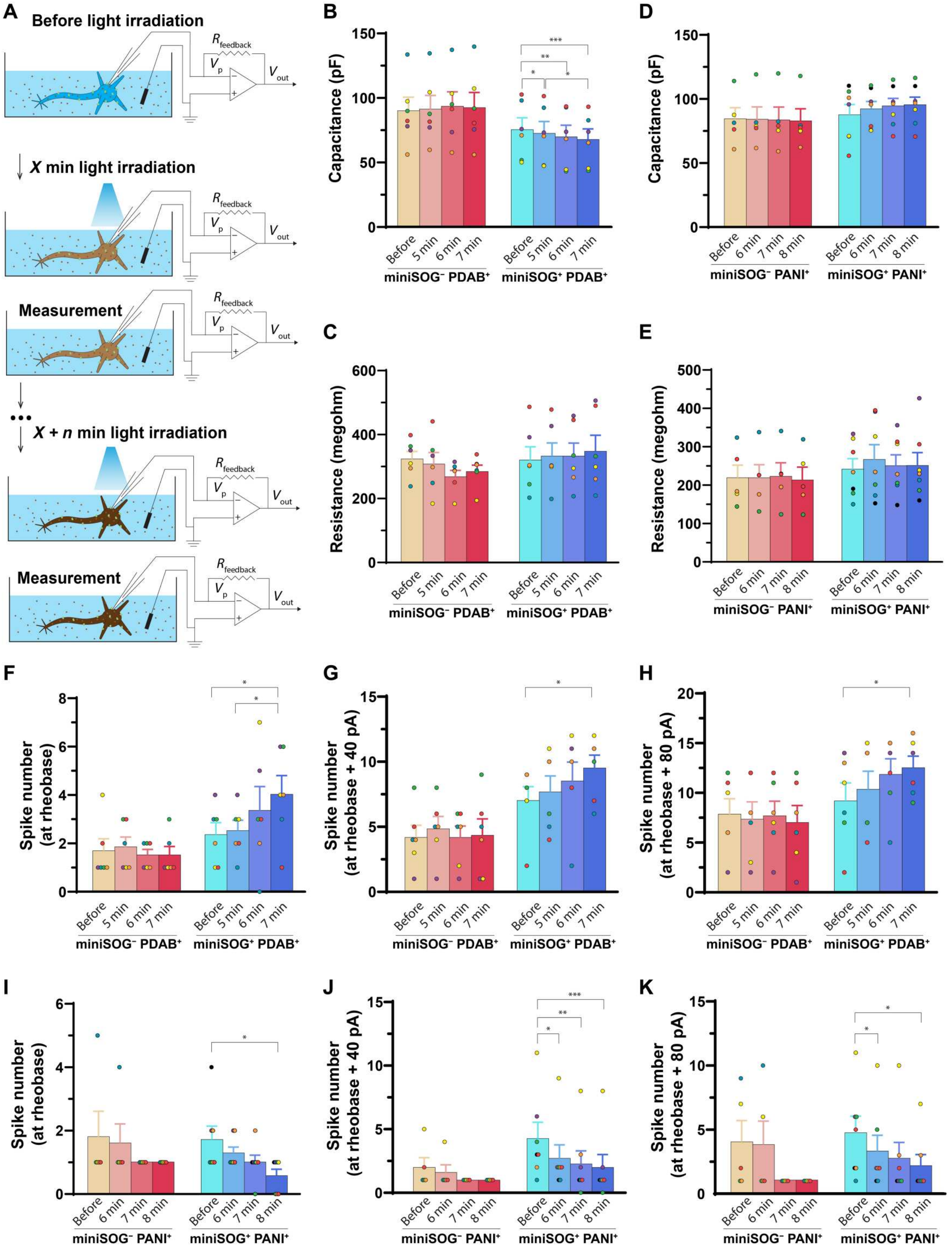


**Fig. 5. Stepwise modulation of single-neuron excitability.** (**A**) Schematics of electrophysiological recording on cultured neurons for change of membrane properties during stepwise polymerization reaction. (**B** and **C**) Change of membrane capacitance and resistance before and after progressively extended blue light irradiation-induced (~5 mW/mm$^2$ for 5, 6, and 7 min) PDAB reaction. (**D** and **E**) Change of membrane capacitance and resistance before and after progressively extended blue light irradiation-induced (~5 mW/mm$^2$ for 6, 7, and 8 min) PANI reaction. (**F** to **H**) Change of spike number at rheobase (F), rheobase + 40 pA (G), and rheobase + 80 pA (H) before and after progressively extended blue light irradiation-induced (~5 mW/mm$^2$ for 5, 6, and 7 min) PDAB reaction. (**I** to **K**) Change of spike number at rheobase (E), rheobase + 40 pA (F), and rheobase + 80 pA (G) before and after progressively extended blue light irradiation-induced (~5 mW/mm$^2$ for 6, 7, and 8 min) PANI reaction. All individual cells were maintained in the whole-cell patch-clamp configuration across pre- and postreaction time points for direct comparison. Bar graphs represent means ± SEM; dots with the same color within each group indicate the same neuron. Paired two tailed *t* test. **P* < 0.05, ***P* < 0.01 and ****P* < 0.001; *t* tests with non-significant *P* values are omitted for clarity.

prefabricated nanomaterials, potentially enabling a new generation of synthetic biology techniques.

## MATERIALS AND METHODS

### Photopolymerization in living and fixed HEK293T cells

HEK293T cells were seeded onto glass coverslips precoated with Matrigel according to the manufacturer's protocol (www.corning.com/worldwide/en/products/life-sciences/resources/webforms/the-ultimate-guide-to-corning-matrigel-matrix.html) and grown in Dulbecco's modified Eagle's medium (DMEM) with 10% fetal bovine serum in a 5% $CO_2$ environment to approximately 90% confluence before transfection. Cells were transfected with either the miniSOG plasmid or the SuperNova Green plasmid under the control of the CAG promoter using Lipofectamine 3000 based on the manufacturer's protocol (www.thermofisher.com/document-connect/document-connect.html?url=https%3A%2F%2Fassets.thermofisher.com%2FTFS-Assets%2FLSG%2Fmanuals%2Flipofectamine3000_protocol.pdf) and then cultured for an additional 24 hours. For living cell experiments, the cells were stained with 10 μM Hoechst 33342 (Thermo Fisher Scientific) for 10 min in FluoroBrite DMEM, rinsed with phosphate-buffered saline (PBS), and then kept in FluoroBrite DMEM until polymerization. During the polymerization, the FluoroBrite DMEM was removed from the well and replaced with the working solution of monomer approximately 5 min before irradiation. Confocal fluorescence and TL images were captured before and after exposure to 475-nm light for 5 to 10 min. For routine photopolymerization, the epifluorescence light source on the GFP filter set was used with the intensity adjusted to approximately 62 $mW/mm^2$.

For fixed cell experiments, the cells were stained with Hoechst as above, rinsed with PBS, and then fixed in 100 mM sodium cacodylate, with 3.2% paraformaldehyde and 0.25% glutaraldehyde (Electron Microscopy Sciences) at pH 7.4 for 15 min before rinsing twice with PBS. Cells were then quenched in 50 mM glycine in PBS for 15 min before rinsing twice again in PBS. The working solution of monomer was prepared as above, except in PBS, and then was added to the cells before performing photopolymerization. Working solutions of the monomer were prepared by dissolving 1 mM DAB or 0.75 to 1 mM aniline + 0.75 to 1 mM PPD in FluoroBrite DMEM, prewarmed and equilibrated in the 5% $CO_2$ environment, and then filtered through a 0.22-μM syringe filter before adding to cells. Stock solutions of DAB or aniline were prepared by dissolving DAB tetrahydrochloride hydrate (Sigma-Aldrich) or aniline (Sigma-Aldrich) to 100 mM in ultrapure water, while the stock solution of PPD (Sigma-Aldrich) was prepared by suspending it to 100 mM in 150 mM HCl. The stock solution was stirred and periodically sonicated at room temperature for 1 to 2 hours to create a fine green-gray suspension that dissolved completely upon dilution into the working solution.

#### *UV/vis spectroscopy of in situ polymerized PANI*

HEK293T cells were cultured and transfected on Aclar coverslips as above. Hoechst staining was skipped, cells were fixed and blocked, and the monomer solution was added as above. To maximize the area exposed to light, polymerization was performed on the 4× objective of an epifluorescence microscope using a 475-nm laser with an approximate intensity of 50 $mW/mm^2$. Cells were then rinsed several times with ultrapure water and air-dried for several hours before acquiring UV/vis spectra on a Cary 60 UV/vis spectrophotometer. For acid doping, the cover slip was kept in a sealed chamber with a few drops of concentrated hydrochloric acid added to the bottom for 1 hour.

#### *Molecular cloning, viral vector construction, and virus production*

For HEK293T expression, the miniSOG coding sequence was inserted into a pAAV-CAG-T2A-dTomato vector backbone for cytosolic expression under the CAG promoter using NEBuilder HiFi DNA assembly. For a superoxide-producing control, the codon-optimized coding sequence of the KillerRed derivative SuperNova Green (*37*), synthesized by GenScript, was inserted instead. For AAV production, the same miniSOG sequence was cloned into a pAAV-hSyn-T2A-mCherry backbone. The following AAV viral vectors under the control of the human synapsin (hSyn) promoter were packaged as AAVdj in our own laboratory: (i) pAAVdj-hSyn-mCherry and (ii) pAAVdj-hSyn-miniSOG-T2A-mCherry. All plasmids used in this paper have been deposited to Addgene.

### Photopolymerization in living and fixed neurons

#### *Primary neurons culture and transfection*

Primary cultures of cortical rat neurons were prepared as follows, following the Institutional Animal Care and Use Committee guidelines. The cortex of Sprague-Dawley rat pups was removed at embryonic day 17. Cortex were digested with papain (0.4 mg/ml) and plated onto 12-mm glass coverslips precoated with 1:80 Matrigel (Corning). Cells were plated in 24-well plates at a density of 100,000 cells per well. The cultured neurons were maintained in NbActiv4 medium (BrainBits) and kept in a humid culture incubator with 5% $CO_2$ at 37°C. Primary culture neurons were infected with $1 \times 10^{10}$ vg of AAVdj-hSyn-miniSOG-T2A-mCherry or AAVdj-hSyn-mCherry at 14 DIV. After 3 to 5 days of expression, photopolymerization was performed according to the above procedures for HEK293T cells.

#### *Biotin-DAB photopolymerization and streptavidin-AF647 staining*

miniSOG$^+$ HEK293T cells or neurons were fixed and glycine-quenched as described above, then permeabilized in 0.1% Triton X-100 in PBS for 15 min, blocked with a streptavidin/biotin blocking kit according to manufacturer's instructions (Thermo Fisher Scientific, cat. no. E21390), and then polymerized as described above for DAB in the presence of 1 mM biotin-DAB instead for 10 min at ~155 $mW/mm^2$ of a 475-nm light. The same was true for neurons, except that the intensity was reduced to ~3.2 $mW/mm^2$. Following polymerization, cells were washed 3× for 5 min with PBS + 0.1% Tween 20 and then stained with a 1:750 dilution of streptavidin-AF647 (Thermo Fisher Scientific, cat. no. S21374) in PBS/Tween 20 for 90 min, followed by 3× 20-min washes of PBS/Tween 20 before confocal imaging.

### Photo-oxidation of PPD with small molecule photosensitizers

A 100 mM suspension of PPD in 150 mM HCl was prepared as described above and then diluted to 500 μM in PBS. Methylene blue (Sigma-Aldrich) or Victoria blue BO (Sigma-Aldrich) was added to a final concentration of 5 μM along with 5 mM sodium azide (Sigma-Aldrich) or superoxide dismutase (45 μg/ml; from bovine erythrocytes; Sigma-Aldrich) from freshly prepared aqueous stock solutions. Reactions were monitored in an untreated, glass-bottom 96-well plate. UV/vis spectra were acquired on a PerkinElmer

EnSpire plate reader before irradiation of the entire well using a 10× microscope objective on the Cy5 Filter Set (~108 mW). After 30 s, no precipitate was visible, and the UV/vis spectra were measured again before continuing irradiation for an additional minute until a dark precipitate began to form in the wells containing methylene blue.

## Electrophysiological characterization

### *Whole-cell patch-clamp*

For whole-cell recording, electrophysiological parameters of cultured neurons were amplified and digitized using the MultiClamp 700B and Digidata1400 (Molecular Devices) and pipettes with a resistance of 4 to 6 megohms filled with an internal solution containing: 128 mM K-gluconate, 10 mM Na-phosphocreatine, 10 mM Hepes, 1.1 mM EGTA, 5 mM adenosine 5′-triphosphate–Mg, and 0.4 mM guanosine 5′-triphosphate–Na with the pH adjusted to 7.4 with KOH. The osmolarity of the internal solution was adjusted to around 300 mosmol with sucrose. The neurons infected with AAVdj-hsyn-mCherry or AAVdj-hsyn-miniSOG-T2A-mCherry cultured on glass coverslips were exposed to Tyrode's solution (150 mM NaCl, 4 mM KCl, 2 mM $MgCl_2$, 2 mM $CaCl_2$, 20 mM glucose, and 10 mM Hepes; titrated to pH 7.35 with NaOH and adjusted osmolarity to 320 to 330 mosmol). 2,3-Dioxo-6-nitro-1,2,3,4-tetrahydrobenzo(*f*)quinoxaline-7-sulfonamide disodium salt (NBQX; 20 μM; Tocris) as the AMPA receptor blocker and D-2-amino-5-phosphono-valeric acid (D-AP5; 25 μM, Tocris) as the *N*-methyl-D-aspartate receptor blocker were contained in all bath solutions. The cells were held in voltage clamp mode at −75 mV. Membrane resistance and cellular capacitance were calculated from a 10-mV depolarization step in voltage clamp. Then, the mode was switched to current clamp followed by stepwise tonic current injection (20 pA per step from −100 to 280 pA) to elicit action potentials and to determine rheobases. Phasic currents (500 pA, 10 ms, and 5 Hz) were injected to generate action potentials while holding at a membrane potential of −70 to −75 mV. Then, the bath solution was switched to the Tyrode's solution containing 1 mM DAB or 0.5 mM aniline + 0.5 mM PPD with NBQX and D-AP5 at pH 7.35 and osmolarity of 320 to 330 mosmol. Recording was repeated after neurons were completely immersed into Tyrode's solution with monomers and after progressively extended blue light irradiation (5, 6, and 7 min stepwise, ~5 $mW/mm^2$) to induce polymerization. Following the end of polymerization, monomers were washed away by replacing the bath solution with Tyrode's solution without monomers, and recording procedure was repeated. Figures 3 and 4 and fig. S10 compared the electrophysiological properties between recorded parameters before adding monomers and washing away monomers. Figure 5 and fig. S12 compared the change of electrophysiological properties when neurons were immersed in the Tyrode's solution containing monomers. Figure S9 compared the electrophysiological properties in the absence of monomers by immersing the neurons in the monomer-free Tyrode's solution during recordings. Analyses of physiological results were performed using Clampfit software (Axon Instruments). Spike width was estimated at the half-peak position from the threshold potential to the peak of a single action potential. Spike latency was estimated as the time point of the peak of an action potential starting from the onset of the current injection. Spike amplitude was estimated as the magnitude of the action potential, taking the resting potential as the baseline. Spike number was estimated by averaging the spike numbers counted at the rheobase, rheobase + 40 pA, and rheobase + 80 pA.

### *Characterization of long-term modulation of neuron excitability and viability*

A total of 1 × $10^5$ primary cultures of cortical rat neurons were added to Nb4 medium and cultured on poly-D-lysine (PDL)-coated glass coverslips. The cultured neurons were infected with 1 × $10^{10}$ vg of AAVdj-hSyn-miniSOG-T2A-mCherry or AAVdj-hSyn-mCherry at 14 DIV, and at 17 DIV, the medium was switched to Tyrode's solution (150 mM NaCl, 4 mM KCl, 2 mM $MgCl_2$, 2 mM $CaCl_2$, 20 mM glucose, and 10 mM Hepes; titrated to pH 7.35 with NaOH and adjusted osmolarity of 320 to 330 mosmol) or Tyrode's solution containing 0.5 mM aniline and 0.5 mM PPD ($PANI^+$) or 1.0 mM DAB ($PDAB^+$). The original Nb4 medium from the cultures was collected and incubated in a humid culture incubator with 5% $CO_2$ at 37°C for later use. The Tyrode's solutions were prefiltered with 0.22-μm filter units. The cultured neurons were then exposed to 7 min of blue light irradiation (~5 $mW/mm^2$) to induce polymerization. Following the end of polymerization, cultured neurons were washed with prewarmed Nb4 medium three times and then maintained in a humid culture incubator with 5% $CO_2$ at 37°C in the original Nb4 medium until subsequent electrophysiological characterization. After additional incubation of 1 or 3 days, the cultured neurons were taken for whole-cell patch-clamp characterization or cell viability assays according to the aforementioned methods.

## Cell viability assay

A total of 2 × $10^4$ primary neurons were added in the Nb4 medium and seeded into each well of 96-well-plate. We divided all the wells into nine groups. They were set as follows: (group 1) $AAV^-$, $monomer^-$, and $irradiation^-$ (baseline control); (group 2) $mCherry^+$, $momoner^-$, and $irradiation^-$ (test effect of AAV infection); (group 3) $miniSOG^+$, $monomer^-$, and $irradiation^-$ (test effect of AAV and miniSOG infection); (group 4) $mCherry^+$, $DAB^-$, and 7-min irradiation; (group 5) $mCherry^+$, $DAB^+$, and 7-min irradiation; (group 6) $mCherry^+$, $PANI^+$, and 7-min irradiation; (group 7) $miniSOG^+$, $DAB^-$, and 7-min irradiation; (group 8) $miniSOG^+$, $DAB^+$, and 7-min irradiation; and (group 9) $miniSOG^+$, $PANI^+$, and 7-min irradiation. At 14 DIV, neurons were infected with 1 × $10^{10}$ vg of AAVdj-hSyn-miniSOG-T2A-mCherry or AAVdj-hSyn-mCherry (except group 1). Three to 5 days after infection when mCherry fluorescence was observed, DAB was added to a final concentration of 1 mM or aniline, and PPD mixture was added to 0.5 mM in Tyrode's solution, respectively, with pH adjusted to 7.3 to 7.4 and osmolarity adjusted to 320 to 330 mosmol. Then, the solution containing monomers was filtered with a 0.22-μm filter unit. Solution containing monomers was added into one of the wells each time following the group settings, and neurons were irradiated for 7 min with ~5 $mW/mm^2$ epifluorescence blue light. The Tyrode's solution was replaced with the original Nb4 media of each well. After all the wells were irradiated and then incubated at 37°C for 30 min for recovery, the cell viability test was then performed. For the cell viability test, the cells were washed one to three times in 1× PBS as needed. Calcein AM (1×)/NucRed Dead 647 ReadyProbes Reagent solution was prepared by diluting the provided calcein AM stocks 1:500 in Nb4 medium and adding two drops of NucRed Dead 647 Reagent per milliliter of medium. The original medium was gently removed from the wells. Each well

was washed three times with fresh Nb4 medium to remove loosely attached and dead cells. A total of 100 μl of medium containing calcein AM and NucRed Dead 647 ReadyProbes Reagent was added to each well. The cells were incubated at room temperature (22° to 26°C) for 5 to 15 min, protected from light. The calcein AM and NucRed solution were removed, and the wells washed two times with fresh Nb4 medium. After the last wash, enough Tyrode's solution was added to cover the cells. The presence of the green calcein AM (excitation of 485 nm and emission of 515 nm) or far-red NucRed Dead 647 ReadyProbes Reagent (excitation of 642 nm and emission of 661 nm) fluorescence in the illuminated area was assessed by fluorescence microscopy using the same imaging parameters for all wells.

### Synthesis and characterization of biotin-DAB

Detailed synthetic methods and characterization can be found in the Supplementary Materials.

## Supplementary Materials

**This PDF file includes:**

Figs. S1 to S15

Supplementary synthetic methods and characterization for Biotin-DAB

## REFERENCES AND NOTES


1. K. S. Cole, *Membranes, Ions and Impulses* (Univ. California Press, 1968).
2. A. L. Hodgkin, A. F. Huxley, A quantitative description of membrane current and its application to conduction and excitation in nerve. *J. Physiol.* **117**, 500–544 (1952).
3. P. Rajasethupathy, E. Ferenczi, K. Deisseroth, Targeting neural circuits. *Cell* **165**, 524–534 (2016).
4. K. Deisseroth, A. Etkin, R. C. Malenka, Optogenetics and the circuit dynamics of psychiatric disease. *JAMA* **313**, 2019–2020 (2015).
5. E. S. Boyden, F. Zhang, E. Bamberg, G. Nagel, K. Deisseroth, Millisecond-timescale, genetically targeted optical control of neural activity. *Nat. Neurosci.* **8**, 1263–1268 (2005).
6. F. Zhang, L.-P. Wang, M. Brauner, J. F. Liewald, K. Kay, N. Watzke, P. G. Wood, E. Bamberg, G. Nagel, A. Gottschalk, K. Deisseroth, Multimodal fast optical interrogation of neural circuitry. *Nature* **446**, 633–639 (2007).
7. O. Yizhar, L. E. Fenno, T. J. Davidson, M. Mogri, K. Deisseroth, Optogenetics in neural systems. *Neuron* **71**, 9–34 (2011).
8. K. Deisseroth, Optogenetics: 10 years of microbial opsins in neuroscience. *Nat. Neurosci.* **18**, 1213–1225 (2015).
9. L. Fenno, O. Yizhar, K. Deisseroth, The development and application of optogenetics. *Annu. Rev. Neurosci.* **34**, 389–412 (2011).
10. F. Zhang, A. M. Aravanis, A. Adamantidis, L. de Lecea, K. Deisseroth, Circuit-breakers: Optical technologies for probing neural signals and systems. *Nat. Rev. Neurosci.* **8**, 577–581 (2007).
11. B. Alberts, R. Heald, A. Johnson, D. Morgan, M. Raff, K. Roberts, P. Walter, J. Wilson, T. Hunt, *Molecular Biology of the Cell* (WW Norton & Company, 2017).
12. A. L. Hodgkin, A. F. Huxley, B. Katz, Measurement of current-voltage relations in the membrane of the giant axon of *Loligo*. *J. Physiol.* **116**, 424–448 (1952).
13. J. Golowasch, F. Nadim, Capacitance, Membrane, in *Encyclopedia of Computational Neuroscience*, D. Jaeger, R. Jung, Eds. (Springer New York, 2013), pp. 1–5.
14. R. Tyzio, A. Ivanov, C. Bernard, G. L. Holmes, Y. Ben-Ari, R. Khazipov, Membrane potential of CA3 hippocampal pyramidal cells during postnatal development. *J. Neurophysiol.* **90**, 2964–2972 (2003).
15. F. Rossi, S. Gianola, L. Corvetti, Regulation of intrinsic neuronal properties for axon growth and regeneration. *Prog. Neurobiol.* **81**, 1–28 (2007).
16. K.-J. Chang, S. A. Redmond, J. R. Chan, Remodeling myelination: Implications for mechanisms of neural plasticity. *Nat. Neurosci.* **19**, 190–197 (2016).
17. M. J. Gillespie, R. B. Stein, The relationship between axon diameter, myelin thickness and conduction velocity during atrophy of mammalian peripheral nerves. *Brain Res.* **259**, 41–56 (1983).
18. G. Matsumoto, I. Tasaki, A study of conduction velocity in nonmyelinated nerve fibers. *Biophys. J.* **20**, 1–13 (1977).
19. B. Howell, L. E. Medina, W. M. Grill, Effects of frequency-dependent membrane capacitance on neural excitability. *J. Neural Eng.* **12**, 056015 (2015).
20. S. H. Weinberg, High-frequency stimulation of excitable cells and networks. *PLOS ONE* **8**, e81402 (2013).
21. S. H. Weinberg, Membrane capacitive memory alters spiking in neurons described by the fractional-order Hodgkin-Huxley model. *PLOS ONE* **10**, e0126629 (2015).
22. S. G. Waxman, Determinants of conduction velocity in myelinated nerve fibers. Muscle nerve off. *Muscle Nerve* **3**, 141–150 (1980).
23. S. G. Waxman, M. V. L. Bennett, Relative conduction velocities of small myelinated and non-myelinated fibres in the central nervous system. *Nat. New Biol.* **238**, 217–219 (1972).
24. F. K. Sanders, D. Whitteridge, Conduction velocity and myelin thickness in regenerating nerve fibres. *J. Physiol.* **105**, 152–174 (1946).
25. R. S. Smith, Z. J. Koles, Myelinated nerve fibers: Computed effect of myelin thickness on conduction velocity. *Am. J. Physiol.* **219**, 1256–1258 (1970).
26. G. Cellot, E. Cilia, S. Cipollone, V. Rancic, A. Sucapane, S. Giordani, L. Gambazzi, H. Markram, M. Grandolfo, D. Scaini, F. Gelain, L. Casalis, M. Prato, M. Giugliano, L. Ballerini, Carbon nanotubes might improve neuronal performance by favouring electrical shortcuts. *Nat. Nanotechnol.* **4**, 126–133 (2009).
27. T. Dvir, B. P. Timko, M. D. Brigham, S. R. Naik, S. S. Karajanagi, O. Levy, H. Jin, K. K. Parker, R. Langer, D. S. Kohane, Nanowired three-dimensional cardiac patches. *Nat. Nanotechnol.* **6**, 720–725 (2011).
28. Y. Jiang, B. Tian, Inorganic semiconductor biointerfaces. *Nat. Rev. Mater.* **3**, 473–490 (2018).
29. M. L. Di Francesco, F. Lodola, E. Colombo, L. Maragliano, M. Bramini, G. M. Paternò, P. Baldelli, M. D. Serra, L. Lunelli, M. Marchioretto, G. Grasselli, S. Cimò, L. Colella, D. Fazzi, F. Ortica, V. Vurro, C. G. Eleftheriou, D. Shmal, J. F. Maya-Vetencourt, C. Bertarelli, G. Lanzani, F. Benfenati, Neuronal firing modulation by a membrane-targeted photoswitch. *Nat. Nanotechnol.* **15**, 296–306 (2020).
30. M. G. Shapiro, K. Homma, S. Villarreal, C.-P. Richter, F. Bezanilla, Infrared light excites cells by changing their electrical capacitance. *Nat. Commun.* **3**, 736 (2012).
31. J. L. Carvalho-de-Souza, J. S. Treger, B. Dang, S. B. H. Kent, D. R. Pepperberg, F. Bezanilla, Photosensitivity of neurons enabled by cell-targeted gold nanoparticles. *Neuron* **86**, 207–217 (2015).
32. C. Sessler, Z. Huang, X. Wang, J. Liu, Functional nanomaterial-enabled synthetic biology. *Nano Futur.* **5**, 022001 (2021).
33. J. Liu, Y. S. Kim, C. E. Richardson, A. Tom, C. Ramakrishnan, F. Birey, T. Katsumata, S. Chen, C. Wang, X. Wang, L.-M. Joubert, Y. Jiang, H. Wang, L. E. Fenno, J. B.-H. Tok, S. P. Paşca, K. Shen, Z. Bao, K. Deisseroth, Genetically targeted chemical assembly of functional materials in living cells, tissues, and animals. *Science* **367**, 1372–1376 (2020).
34. S. S. Lam, J. D. Martell, K. J. Kamer, T. J. Deerinck, M. H. Ellisman, V. K. Mootha, A. Y. Ting, Directed evolution of APEX2 for electron microscopy and proximity labeling. *Nat. Methods* **12**, 51–54 (2014).
35. X. Shu, V. Lev-Ram, T. J. Deerinck, Y. Qi, E. B. Ramko, M. W. Davidson, Y. Jin, M. H. Ellisman, R. Y. Tsien, A genetically encoded tag for correlated light and electron microscopy of intact cells, tissues, and organisms. *PLoS Biol.* **9**, e1001041 (2011).
36. S. Pletnev, N. G. Gurskaya, N. V. Pletneva, K. A. Lukyanov, D. M. Chudakov, V. I. Martynov, V. O. Popov, M. V. Kovalchuk, A. Wlodawer, Z. Dauter, V. Pletnev, Structural basis for phototoxicity of the genetically encoded photosensitizer KillerRed. *J. Biol. Chem.* **284**, 32028–32039 (2009).
37. Y. D. Riani, T. Matsuda, K. Takemoto, T. Nagai, Green monomeric photosensitizing fluorescent protein for photo-inducible protein inactivation and cell ablation. *BMC Biol.* **16**, 50 (2018).
38. R. Ruiz-González, A. L. Cortajarena, S. H. Mejias, M. Agut, S. Nonell, C. Flors, Singlet oxygen generation by the genetically encoded tag miniSOG. *J. Am. Chem. Soc.* **135**, 9564–9567 (2013).
39. J. N. Demas, E. W. Harris, R. P. McBride, Energy transfer from luminescent transition metal complexes to oxygen. *J. Am. Chem. Soc.* **99**, 3547–3551 (1977).
40. K. Teshima, K. Yamada, N. Kobayashi, R. Hirohashi, Photopolymerization of aniline with a tris (2, 2′-bipyridyl) ruthenium complex-methylviologen polymer bilayer electrode system. *Chem. Commun.* , 829–830 (1996).
41. S. Uemura, K. Teshima, S. Tokuda, N. Kobayashi, R. Hirohashi, Photopolymerized conducting potyaniline micropattern and its application. *Synth. Met.* **101**, 701–702 (1999).
42. K. Briviba, T. P. A. Devasagayam, H. Sies, S. Steenken, Selective para hydroxylation of phenol and aniline by singlet molecular oxygen. *Chem. Res. Toxicol.* **6**, 548–553 (1993).
43. J. Al-Nu'airat, M. Altarawneh, X. Gao, P. R. Westmoreland, B. Z. Dlugogorski, Reaction of aniline with singlet oxygen ($O_2{}^1\Delta_g$). *J. Phys. Chem. A* **121**, 3199–3206 (2017).
44. A. P. Darmanyan, W. S. Jenks, P. Jardon, Charge-transfer quenching of singlet oxygen $O_2(^1\Delta_g)$ by amines and aromatic hydrocarbons. *J. Phys. Chem. A* **102**, 7420–7426 (1998).
45. M. Łapkowski, Electrochemical synthesis of linear polyaniline in aqueous solutions. *Synth. Met.* **35**, 169–182 (1990).

46. E. M. Genies, M. Lapkowski, Spectroelectrochemical evidence for an intermediate in the electropolymerization of aniline. *J. Electroanal. Chem. Interfacial Electrochem.* **236**, 189–197 (1987).
47. E. Skovsen, J. W. Snyder, J. D. C. Lambert, P. R. Ogilby, Lifetime and diffusion of singlet oxygen in a cell. *J. Phys. Chem. B* **109**, 8570–8573 (2005).
48. J. Y. Lin, S. B. Sann, K. Zhou, S. Nabavi, C. D. Proulx, R. Malinow, Y. Jin, R. Y. Tsien, Optogenetic inhibition of synaptic release with chromophore-assisted light inactivation (CALI). *Neuron* **79**, 241–253 (2013).
49. E. A. Souslova, K. E. Mironova, S. M. Deyev, Applications of genetically encoded photosensitizer miniSOG: From correlative light electron microscopy to immunophotosensitizing. *J. Biophotonics* **10**, 338–352 (2017).
50. J. E. Natera, W. A. Massad, F. Amat-Guerri, N. A. García, Elementary processes in the eosin-sensitized photooxidation of 3,3′-diaminobenzidine for correlative fluorescence and electron microscopy. *J. Photochem. Photobiol. A Chem.* **220**, 25–30 (2011).
51. S. E. Moulton, P. C. Innis, L. A. P. Kane-Maguire, O. Ngamna, G. G. Wallace, Polymerisation and characterisation of conducting polyaniline nanoparticle dispersions. *Curr. Appl. Phys.* **4**, 402–406 (2004).
52. S. Xu, A. D. Chisholm, Highly efficient optogenetic cell ablation in *C. elegans* using membrane-targeted miniSOG. *Sci. Rep.* **6**, 21271 (2016).
53. Y. B. Qi, E. J. Garren, X. Shu, R. Y. Tsien, Y. Jin, Photo-inducible cell ablation in *Caenorhabditis elegans* using the genetically encoded singlet oxygen generating protein miniSOG. *Proc. Natl. Acad. Sci. U.S.A.* **109**, 7499–7504 (2012).
54. M. A. Dichter, Rat cortical neurons in cell culture: Culture methods, cell morphology, electrophysiology, and synapse formation. *Brain Res.* **149**, 279–293 (1978).
55. T. Breitenbach, P. R. Ogilby, J. D. C. Lambert, Effect of intracellular photosensitized singlet oxygen production on the electrophysiological properties of cultured rat hippocampal neurons. *Photochem. Photobiol. Sci.* **9**, 1621–1633 (2010).
56. D. Mezdour, Dielectric properties of polyaniline composites. *Spectrosc. Lett.* **50**, 214–219 (2017).
57. M. Gray, K. M. Lett, V. B. Garcia, C. W. Kyi, K. A. Pennington, L. C. Schulz, D. J. Schulz, Changes in excitability and ion channel expression in neurons of the major pelvic ganglion in female type II diabetic mice. *Auton. Neurosci.* **220**, 102558 (2019).
58. P. J. Courtoy, D. H. Picton, M. G. Farquhar, Resolution and limitations of the immunoperoxidase procedure in the localization of extracellular matrix antigens. *J. Histochem. Cytochem.* **31**, 945–951 (1983).
59. J. He, Y. Wang, M. A. Missinato, E. Onuoha, L. A. Perkins, S. C. Watkins, C. M. S. Croix, M. Tsang, M. P. Bruchez, A genetically targetable near-infrared photosensitizer. *Nat. Methods* **13**, 263–268 (2016).

**Acknowledgments**
**Funding:** J.L. acknowledges the support from the Air Force Office of Scientific Research (AFOSR) Young Investigator Program (YIP) award no. FA9550-22-1-0228, the NSF through the Harvard University Materials Research Science and Engineering Center grant no. DMR-2011754, and the Harvard Dean's Competitive Fund for Promising Scholarship. X.W. acknowledge the supports from Stanley Center gift and Thomas D. and Virginia W. Cabot assistant professorship. **Author contributions:** J.L., X.W., C.D.S., and Y.Z. conceived the ideas. C.D.S. developed and optimized the photosensitizer protein-based polymerization reactions. Y.Z. and W.W. performed the whole-cell patch-clamp characterization. Y.Z. performed the cell viability tests. W.W. synthesized the molecules for polymer labeling. N.D.H. and Z.F. provided insight and built and maintained the patch-clamp recording platform. D.G. and M.S. provided the primary neuron cultures. C.D.S., Y.Z., X.W., and J.L. wrote the manuscript with input from all authors. J.L. and X.W. supervised the project. **Competing interests:** M.S. serves on the Scientific Advisory Board of Biogen, Cerevel, Neumora, Vanqua Bio, and ArcLight Therapeutics. A patent application related to this work is submitted by the Broad Institute. C.D.S., Y.Z., X.W., and J.L. are coinventors on a provisional patent application related to this work to be filed by Broad Institute of MIT and Harvard. The authors declare that they have no other competing interests. **Data and materials availability:** All data needed to evaluate the conclusions in the paper are present in the paper and/or the Supplementary Materials.

Submitted 26 July 2022
Accepted 28 October 2022
Published 7 December 2022
10.1126/sciadv.ade1136
