# Supplementary material for: Optogenetic polymerization and assembly of electrically functional polymers for modulation of single-neuron excitability
Source: Sci Adv. 2022 Dec 7;8(49):eade1136. doi: 10.1126/sciadv.ade1136 (PMC9728971; doi:10.1126/sciadv.ade1136)
Supplement: Supplementary file 3 — Erratum published 30 June 2023: The original version of the Research Article contained an error in supplemental figure S8. In figure S8, the image for the AAV-/monomer-/irradiation- condition was a duplicate of the image used for the miniSOG+/monomer-/irradiation- condition. The image for the AAV-/monomer-/irradiation- condition has been replaced with the correct version. In the previous version of figure S8, the labels for the representative images in panel B were incorrect. The labels in figure S8B have been rearranged for greater clarity. In the caption for figure S4B, the duration of exposure was changed from 5 to 7 minutes. The online PDF, Supplementary Materials PDF, and HTML (full text) have been updated. The original version is available here: [file sciadv.ade1136_sm.v1.pdf]

Science Advances
AAAS

Supplementary Materials for

Optogenetic polymerization and assembly of electrically functional polymers for modulation of single-neuron excitability

Chanan D. Sessler *et al.*

Corresponding author: Jia Liu, jia_liu@seas.harvard.edu; Xiao Wang, xwangx@mit.edu

Sci. Adv. **8**, eade1136 (2022)
DOI: 10.1126/sciadv.ade1136

This PDF file includes:

Figs. S1 to S15
Supplementary synthetic methods and characterization for Biotin-DAB

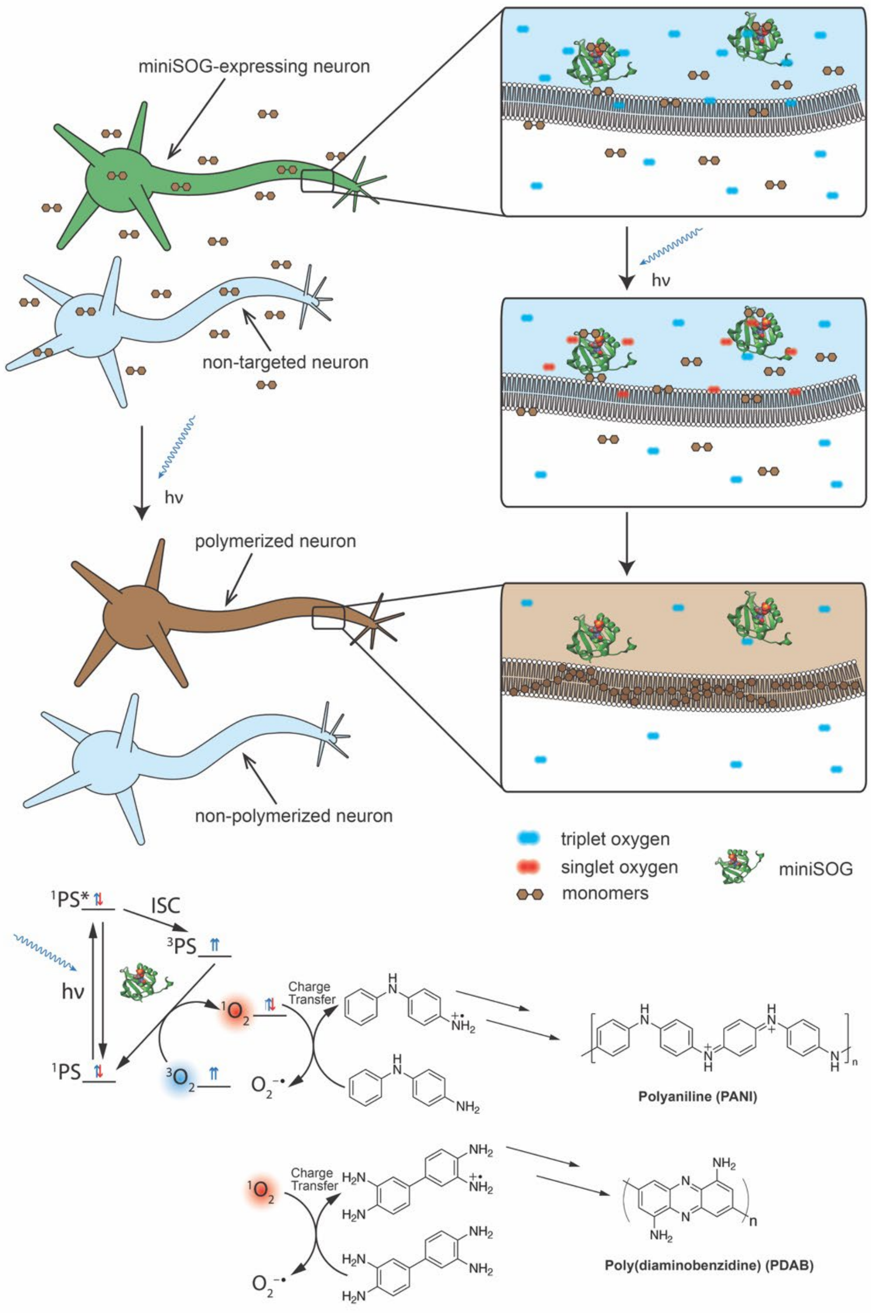

Figure S1. Proposed molecular mechanism of miniSOG-catalyzed optogenetic polymerization. Irradiation of miniSOG (green ribbon model) converts triplet oxygen (blue spheres) to its first excited, singlet state (red spheres). In the presence of reactive monomers (brown

hexagons) singlet oxygen is rapidly quenched through a charge transfer complex, generating aminium radical cations, which subsequently polymerize and precipitate onto or within the membrane (brown aggregates) once a sufficient chain length is reached.

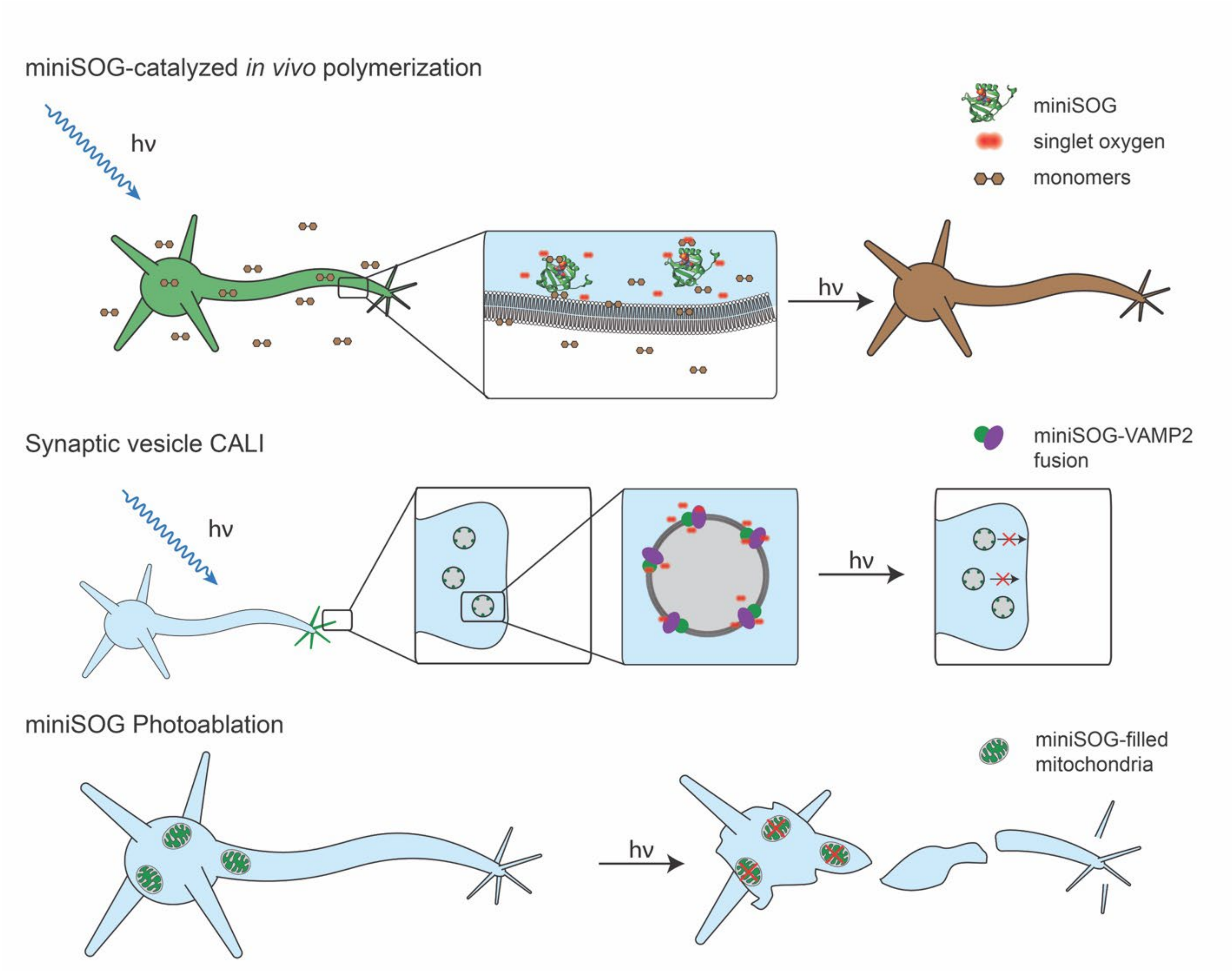

Figure S2. Comparison of cell toxicity among miniSOG-catalyzed optogenetic polymerization, chromophore assisted light inactivation, and photoablation. Top: In the technique discussed in this manuscript, singlet oxygen (red spheres) generated by miniSOG (green) upon irradiation quickly reacts with relatively high (1 mM) concentrations of quenching monomers (brown hexagons), coating the cell with functional polymers. Middle: In the previous example of miniSOG CALI targeting synaptic vesicle proteins, miniSOG is fused with VAMP2 or synaptophysin (purple ovals) (*48*). Singlet oxygen reacts with amino acid side chains in both the fused protein and its interactors, deactivating them, and preventing synaptic vesicle fusion. Bottom: In the previous example of miniSOG photoablation, miniSOG is targeted to the mitochondrial matrix, disrupting cellular respiration and ultimately leading to complete cell death[53].

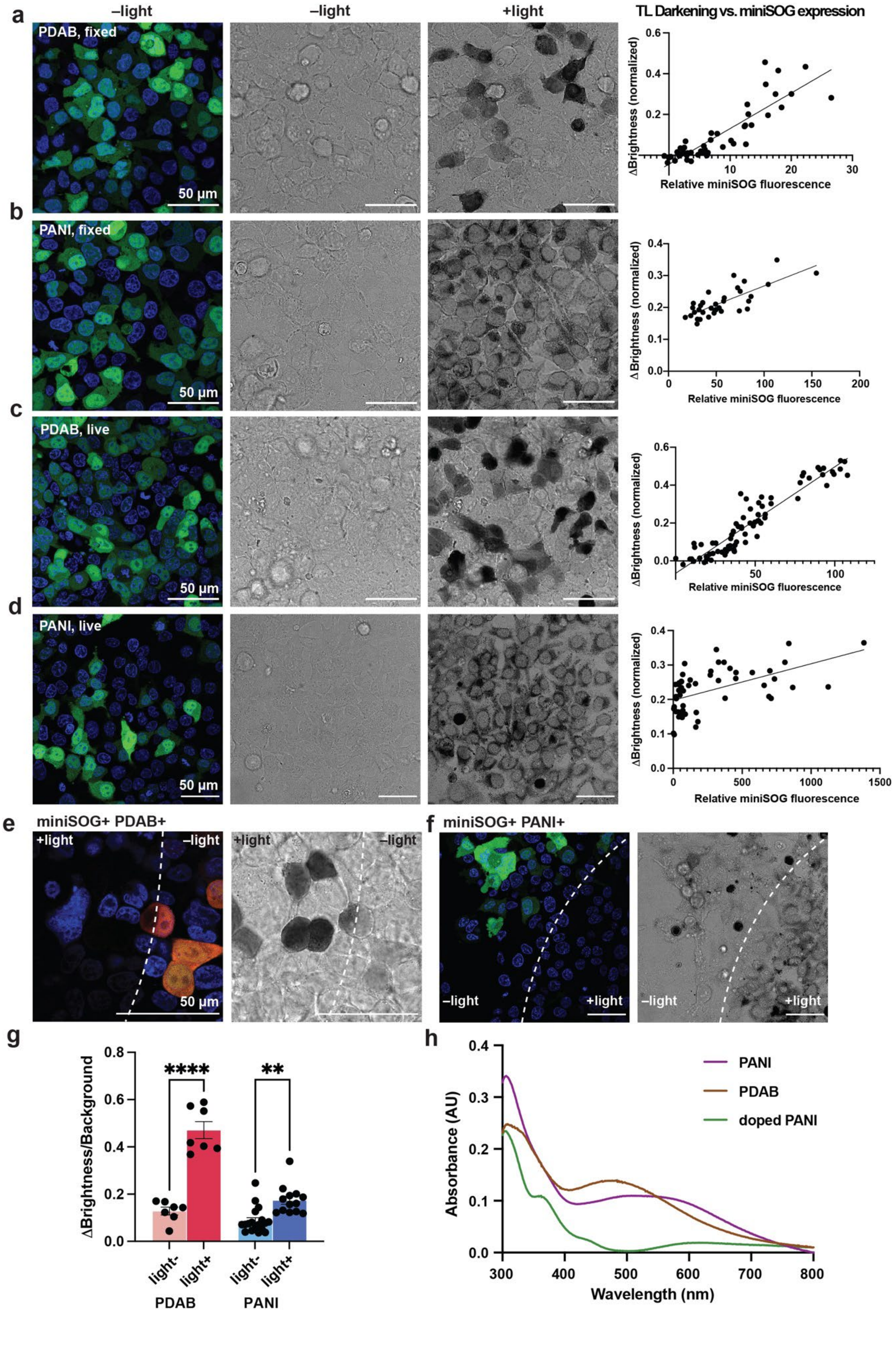
a
–light
–light
+light
TL Darkening vs. miniSOG expression
PDAB, fixed
50 µm
ΔBrightness (normalized)
Relative miniSOG fluorescence
b
PANI, fixed
50 µm
c
PDAB, live
50 µm
d
PANI, live
50 µm
e
miniSOG+ PDAB+
+light
–light
50 µm
f
miniSOG+ PANI+
–light
+light
g
ΔBrightness/Background
****
**
light-
light+
PDAB
PANI
h
Absorbance (AU)
Wavelength (nm)
PANI
PDAB
doped PANI

Figure S3. Spatial specificity of PANI and PDAB optogenetic polymerization. **a-d**, From left to right: fluorescence images showing fluorescence of miniSOG (green) and Hoechst 33342 (blue) in fixed (**a, b**) and living (**c, d**) HEK cells prior to light exposure; TL images showing the cells before and after polymerization of PDAB (**a, c**) and PANI (**b, d**); Correlation between initial miniSOG fluorescence and darkening of cells in TL image after irradiation. Normalized fluorescence and TL brightness were calculated as described in **Figure 1**. $R^2 = 0.77$, $p < 0.0001$ (**a**); $R^2 = 0.58$, $p < 0.0001$ (**b**). $R^2 = 0.89$, $p < 0.0001$ (**c**); and $R^2 = 0.30$, $p < 0.0001$ (**d**). **e-f,** light-patterned optogenetic polymerization of DAB (**e**) and PANI (**f**) in living HEK293T cells. Images represent the boundary of the light-irradiated area from the objective as in **Figure 1**. **g**, Bar and dot plots showing the level of the polymerization, quantified as in **Figure 1d**; unpaired, two-tailed t-tests, ** $p < 0.01$, **** $p < 0.0001$. **h**, Representative UV/Vis spectra of PDAB, PANI, and hydrochloric acid-doped PANI on miniSOG+ HEK293T cells

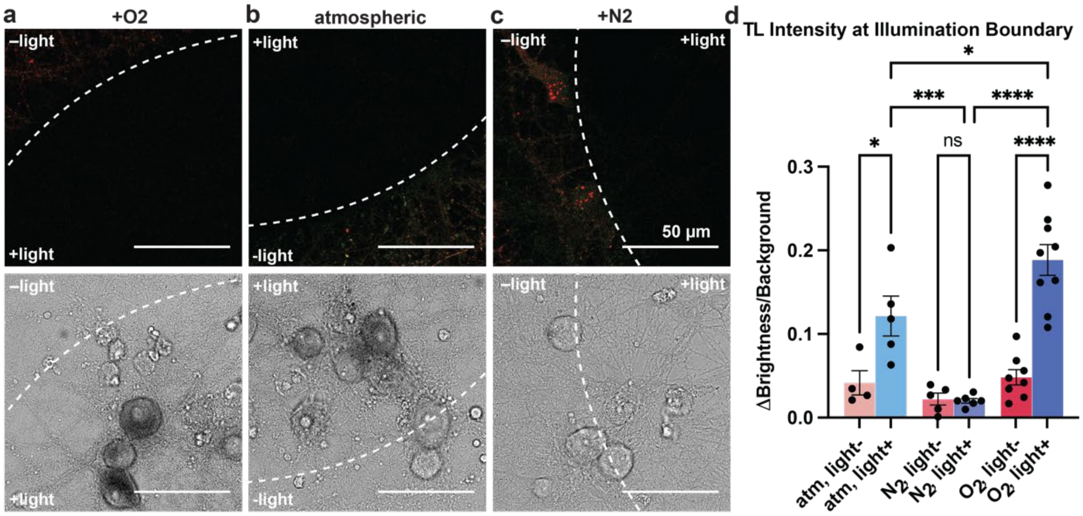

Figure S4. Optogenetic polymerization of PANI in the presence and absence of oxygen. **a-c**, miniSOG-catalyzed polymerizations in neurons under different conditions. Neurons were fixed and treated with the monomer solution as described in the methods section. A thin stream of medical grade oxygen (**a**) or nitrogen (**c**) was bubbled through the monomer solution for 10-15 minutes immediately prior to polymerization, compared to monomer solution treated with neither gas (**b**). Fluorescence (green: miniSOG and red: mCherry) and TL images are shown at the boundary of irradiation after being exposed to the GFP filter set light source for 5 minutes). **d,** Relative darkening of cells in TL images, quantified as in **Figure 1i-j**. *$p < 0.05$; ***$p < 0.001$, ****$p < 0.0001$.

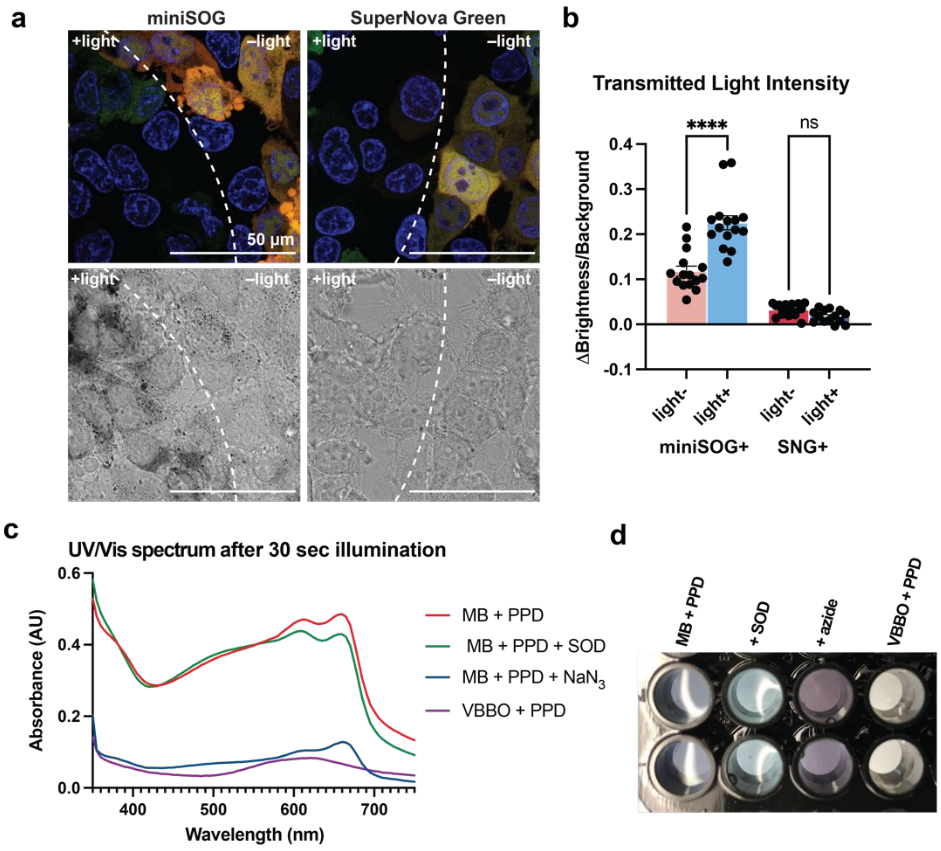

Figure S5. Optogenetic polymerization of PANI with singlet oxygen or radical producing photosensitizers. a, Fluorescence images show Hoechst (blue) miniSOG (green) or the superoxide producing photosensitizing protein SuperNova Green (green) and mCherry (red). Images at the irradiation boundary were collected 10 minutes after irradiation. Blebbing of irradiated, SNG+ cells is noticeable, consistent with photoablation by the production of radical ROS in these cells. **b**, Quantification of polymerization as in **Figure 1d**. **c**, *In vitro* polymerization reactions were prepared in the dark, UV/vis spectra were acquired, then each well was irradiated with the same epifluorescence microscope light source using a standard Cy5 filter set for 30 sec, and the UV/vis spectra acquired again before significant insoluble precipitates formed. The wells were irradiated for another 30 seconds before collecting the image in panel. **d**, MB: 5 µM methylene blue, a singlet oxygen producing photosensitizer; PPD: 500 µM aniline dimer; SOD: 45 µg/mL superoxide dismutase; NaN_3: 5 mM sodium azide, a selective singlet oxygen quencher; VBBO: 5 µM Victoria Blue BO, a superoxide producing photosensitizer. ****$p < 0.0001$.

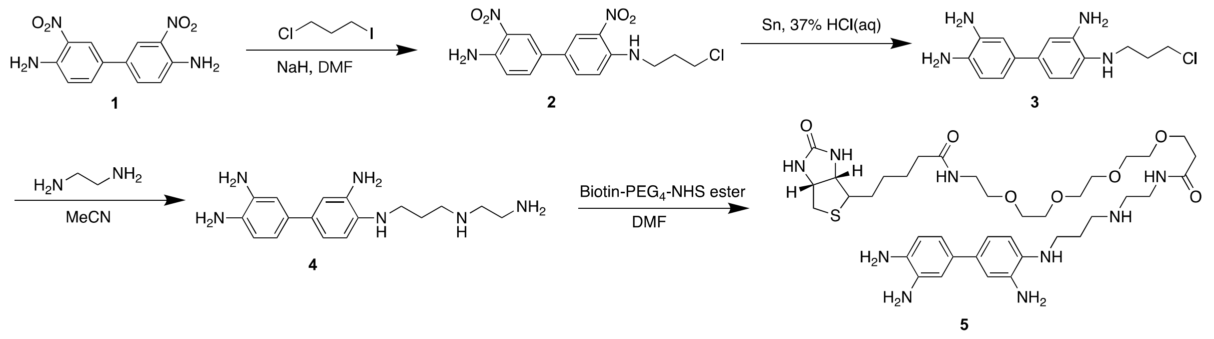

Figure S6. Synthetic scheme for biotin-DAB.

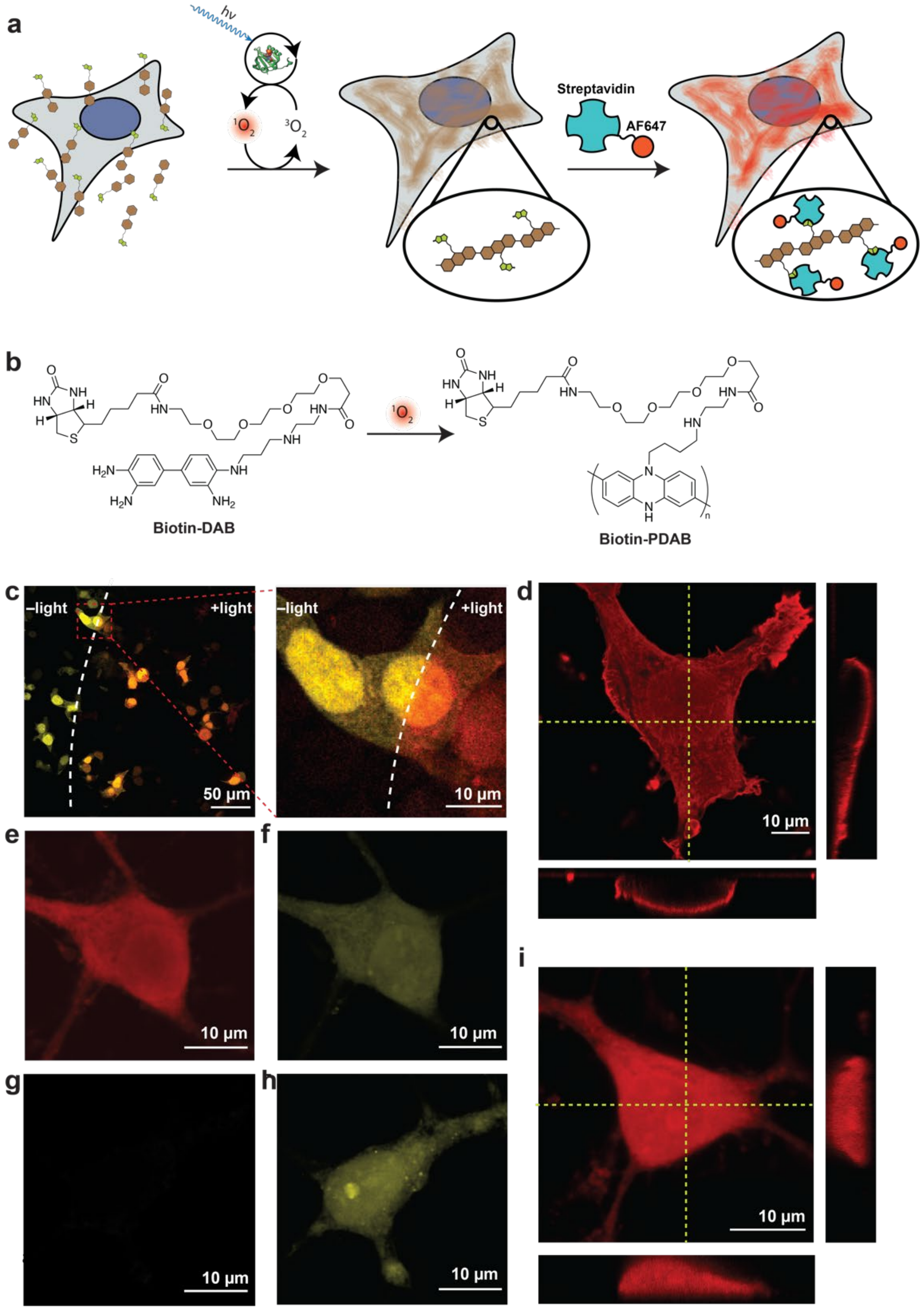

Figure S7. Imaging of optogenetically patterned polymers at subcellular resolution. a, Schematics of methods for labeling polymers inside cells. Biotin-DAB is polymerized under the same condition as that used for cell viability and whole-cell patch-clamp measurement. Subsequently, the remaining monomers are washed out, and the biotin-containing PDAB is stained with a streptavidin-Alexa Fluor (AF) 647 conjugate. **b**, Structures of biotin-DAB and corresponding polymer. **c**, Merged confocal fluorescence images of fixed HEK293T cells co-

expressing miniSOG and dTomato (yellow), irradiated in the presence of 1 mM biotin-DAB, followed by staining with streptavidin-AF647 (red). **d**, Maximum intensity Z projection of streptavidin-AF647 staining in a polymerized and stained HEK293T cell. Yellow dashed lines represent the re-slicing sections (right and bottom panels) along the x- and y-axis. **e-g**, Confocal fluorescence images of AF647 and mCherry of representative miniSOG+ (**e-f**) and miniSOG– (**g-h**) neurons after irradiation. The expression level was controlled to be similar to those used for cell viability and whole-cell patch-clamp characterizations: ~3.2 mW/mm^2 irradiation of 475 nm light in the presence of 1 mM biotin-DAB followed by streptavidin-AF647 staining. **i**, Maximum intensity Z projection of streptavidin-AF647 staining in a polymerized and stained neuron. Yellow dashed lines represent the re-slicing sections (right and bottom panels) along the x- and y-axis.

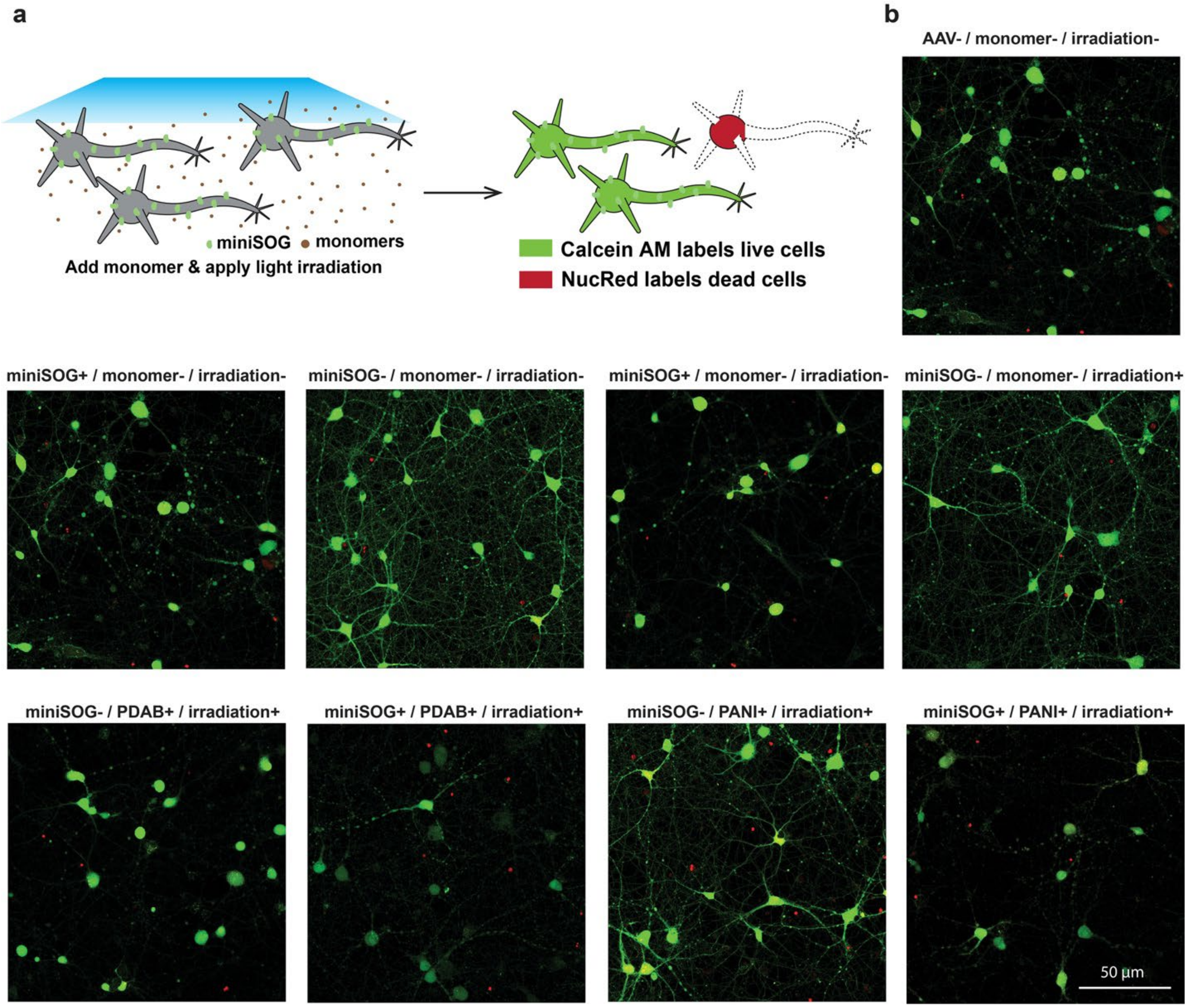

Figure S8. **Assessment of cell viability after polymerization. a**, Schematics illustrating the optogenetic polymerization in living cells and staining for cell viability test. **b**, Representative images of acute cell viability tested immediately after polymerization reaction. 9 different groups are assessed. Light intensities used for polymerization are approximately 5 mW/mm^2

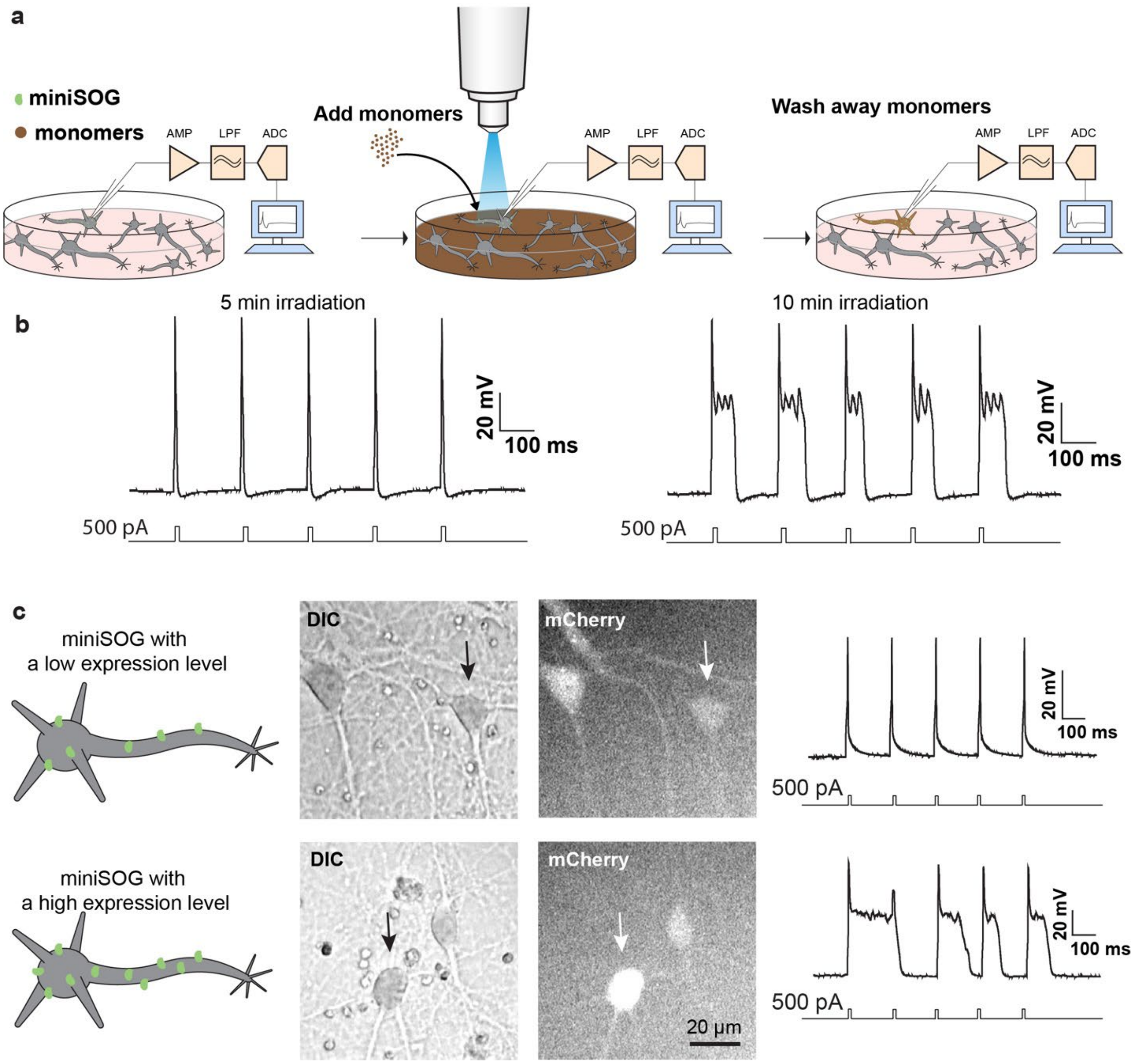

Figure S9. Controlling irradiation time and expression level of miniSOG to avoid neuronal hyperexcitability. **a**, Schematics of the continuous whole-cell patch-clamp characterization on cultured primary neurons to optimize the reaction condition. The setup for whole-cell recording includes an amplifier (AMP), a low pass filter (LPF) and an analogue-to-digital converter (ADC). **b**, Representative traces evoked by phasic currents (500 pA, 10 ms, 5 Hz) injection after 5 min (short) and 10 min (long) irradiation. **c**, Neurons with the weak or high expression level of miniSOG are determined and selected based on the fluorescence intensity for the whole-cell patch-clamp characterization. Light intensities used for the optogenetic polymerization are approximately 5 mW/mm^2. Light intensities used for mCherry imaging are approximately 7 mW/mm^2

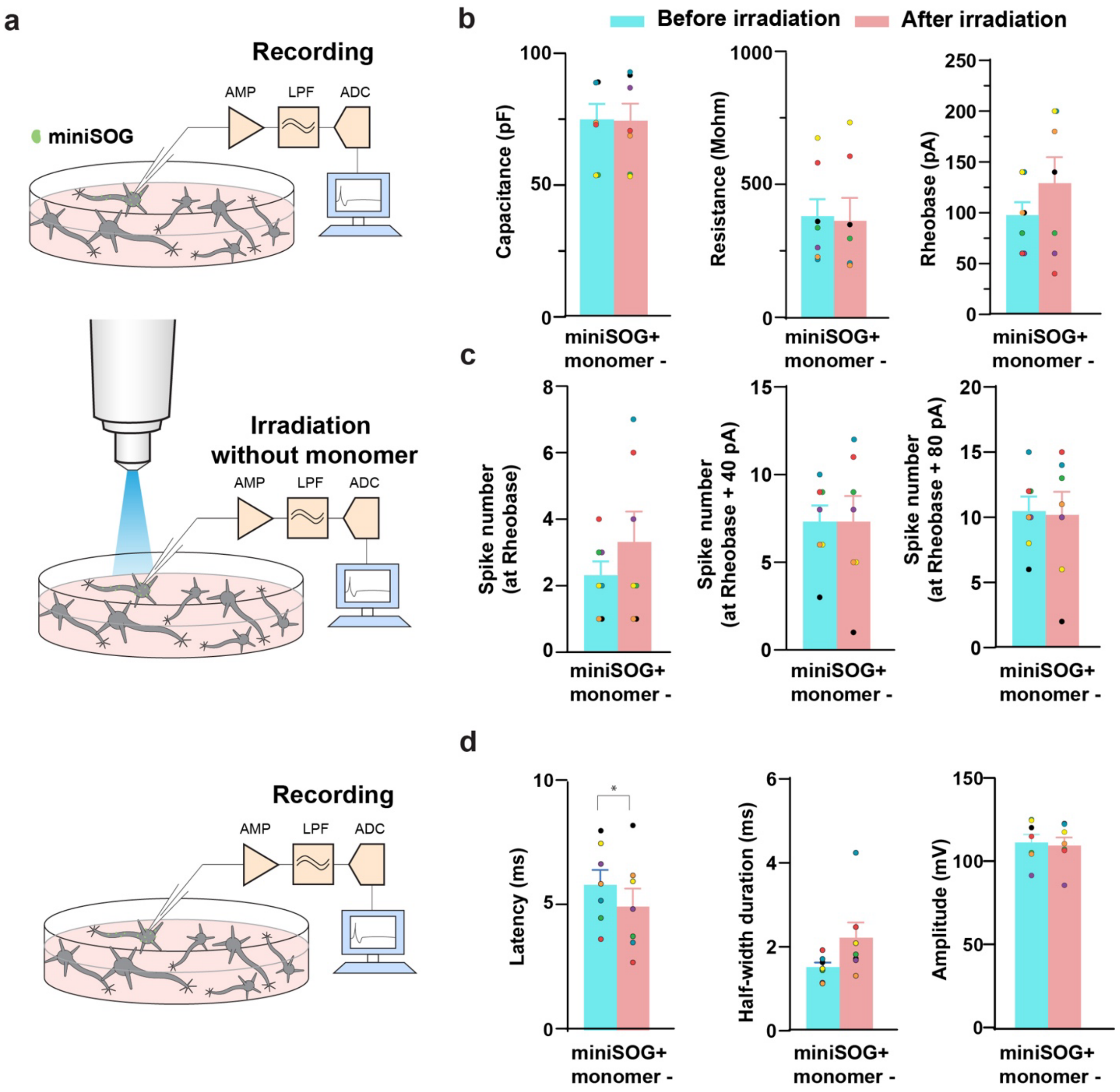

Figure S10. Membrane properties of neurons before and after light irradiation in the absence of DAB. a, Schematics of the continuous whole-cell patch-clamp characterization on cultured primary neurons before and after light irradiation for the characterization of membrane property and excitability. **b**, Membrane capacitance and membrane resistance measured by 10 mV hyperpolarization step in voltage clamp. The rheobase was determined by stepwise tonic current injection. Neurons were irradiated by blue light with the same intensity and duration as **Figure 3** (5 mW/mm^2, 7 minutes). **c**, Statistics of the stepwise tonic current-injection-evoked spikes at rheobase, rheobase+40 pA, and rheobase+80 pA in current clamp before and after light irradiation. **d**, Latency, half-width duration, and amplitude extracted from spikes evoked by phasic currents (500 pA, 10 ms, 5 Hz) injection. All individual cells were successfully maintained in the whole-cell patch-clamp configuration before and after the irradiation for direct comparison. Bar graphs

represent mean ± s.e.m. dots with the same color within each group indicate the same neuron. Paired two tailed t-test. *$p < 0.05$.

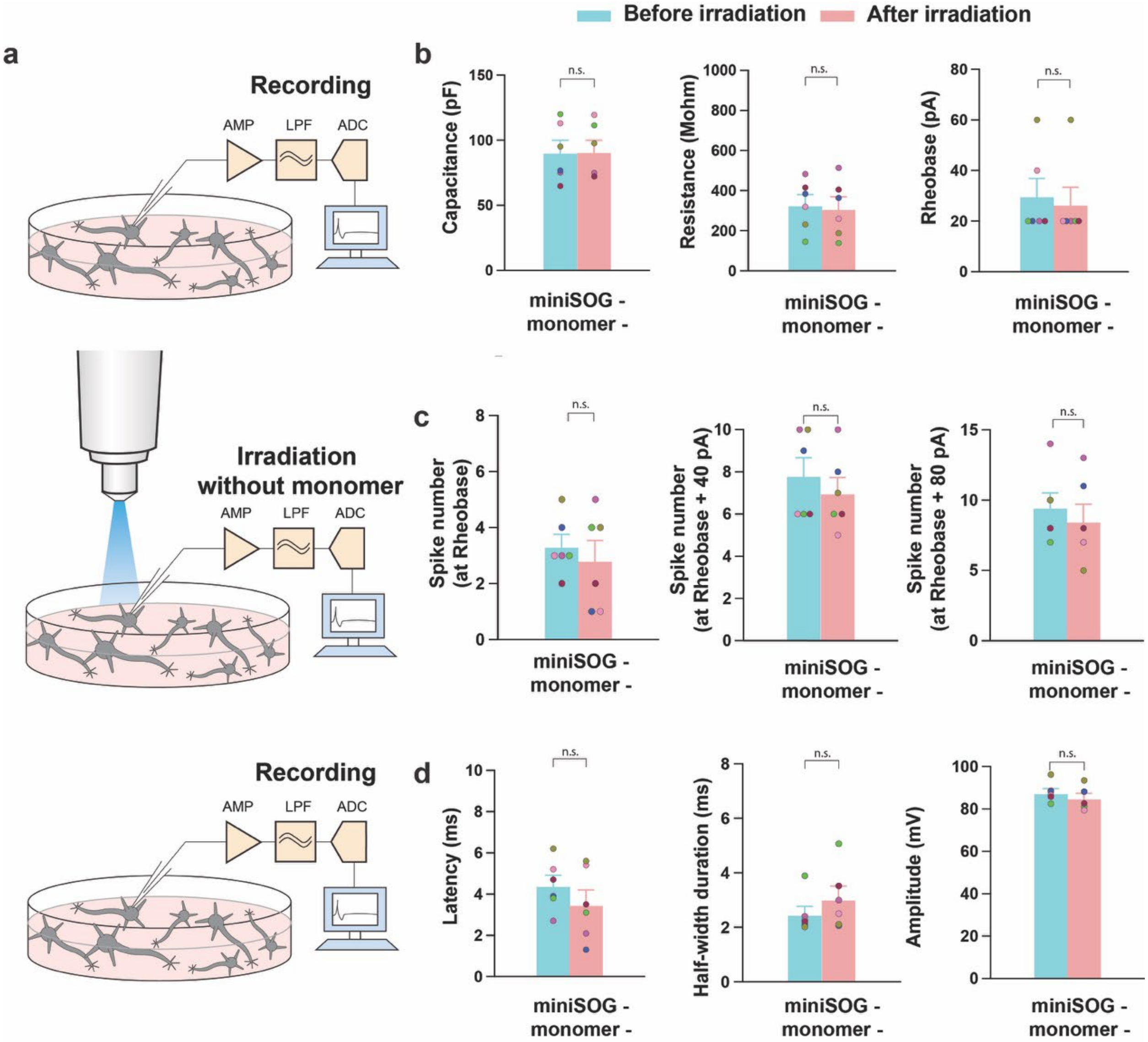

Supplementary Figure 11. Membrane properties of miniSOG- neurons before and after light irradiation in the absence of monomers. a, Schematics of the continuous whole-cell patch-clamp characterization on miniSOG- cultured primary neurons before and after light irradiation for the characterization of membrane property and excitability. **b**, Membrane capacitance and membrane resistance measured by 10 mV hyperpolarization step in voltage clamp. The rheobase was determined by stepwise tonic current injection. Neurons were irradiated by blue light with the same intensity and duration as **Figure 3** (5 mW/mm^2, 7 minutes). **c**, Statistics of the stepwise tonic current-injection-evoked spikes at rheobase, rheobase + 40 pA, and rheobase + 80 pA in current clamp before and after light irradiation. **d**, Latency, half-width duration, and amplitude extracted from spikes evoked by phasic currents (500 pA, 10 ms, 5 Hz) injection. All individual cells were successfully maintained in the whole-cell patch-clamp configuration before and after the irradiation for direct comparison. Bar graphs represent mean ± s.e.m. dots with the same color within each group indicate the same neuron. Paired two tailed t-test. *$p < 0.05$.

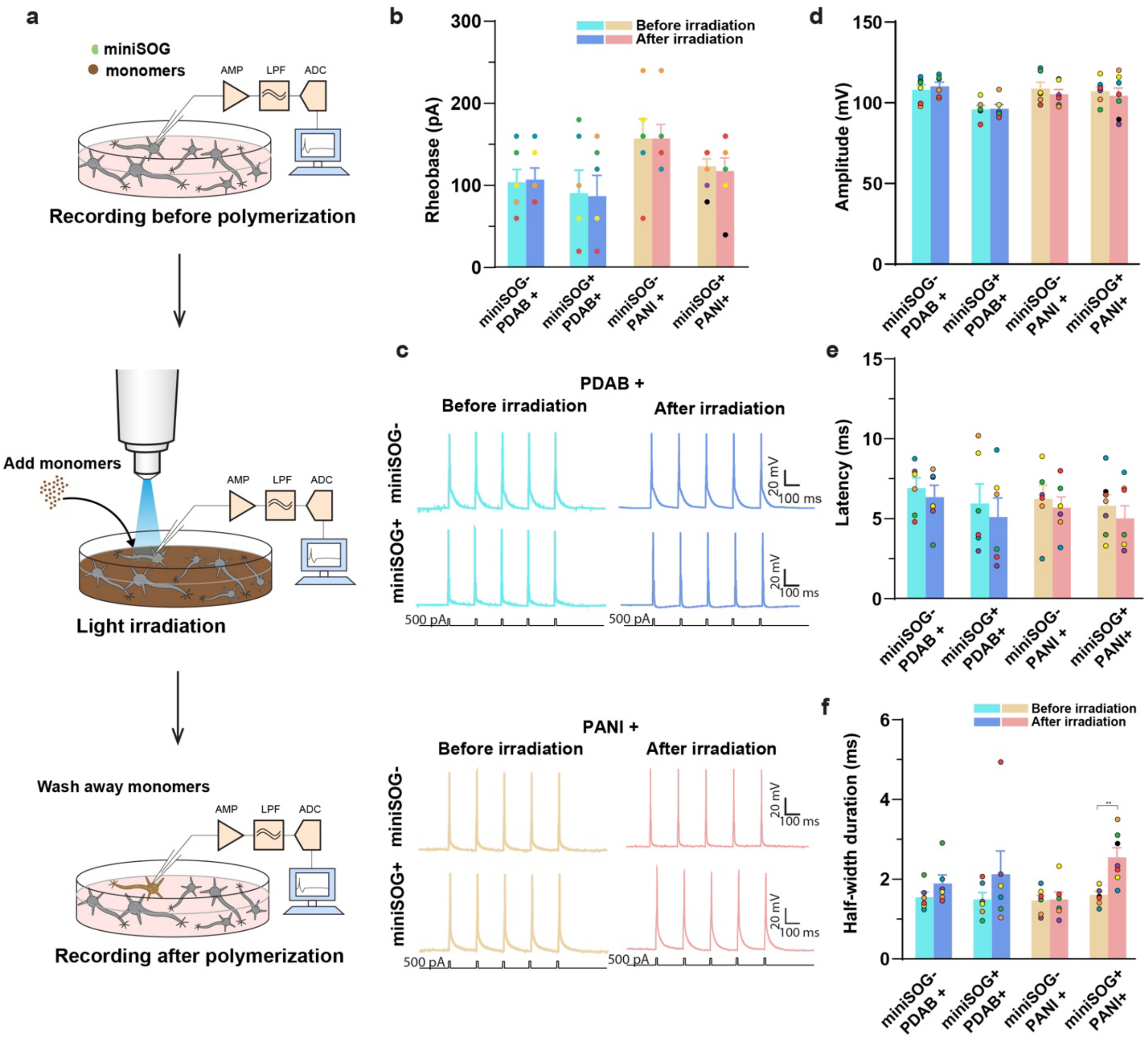

Supplementary Figure 12. Electrophysiological characterization of neurons before and after optogenetic polymerization of PDAB and PANI. a, Workflow of the continuous whole-cell patch-clamp characterization on cultured primary neurons before and after light irradiation for membrane kinetics measurement. **b**, Rheobase determined by stepwise tonic current injection. **c**, Representative traces evoked by phasic currents (500 pA, 10 ms, 5 Hz) injection. **d-f**, Amplitude (**d**), latency (**e**) and half-width duration (**f**) extracted from spikes evoked by phasic currents (500 pA, 10 ms, 5 Hz) injection. All individual cells were maintained in the whole cell patch clamp configuration across pre-reaction and post-reaction time points for direct comparison. Bar graphs represent mean ± s.e.m, dots with the same color within each group indicate the same neuron. Paired two tailed t-test. **$p < 0.01$ versus group of before irradiation. Light intensities used for polymerization are approximately 5 mW/mm^2.

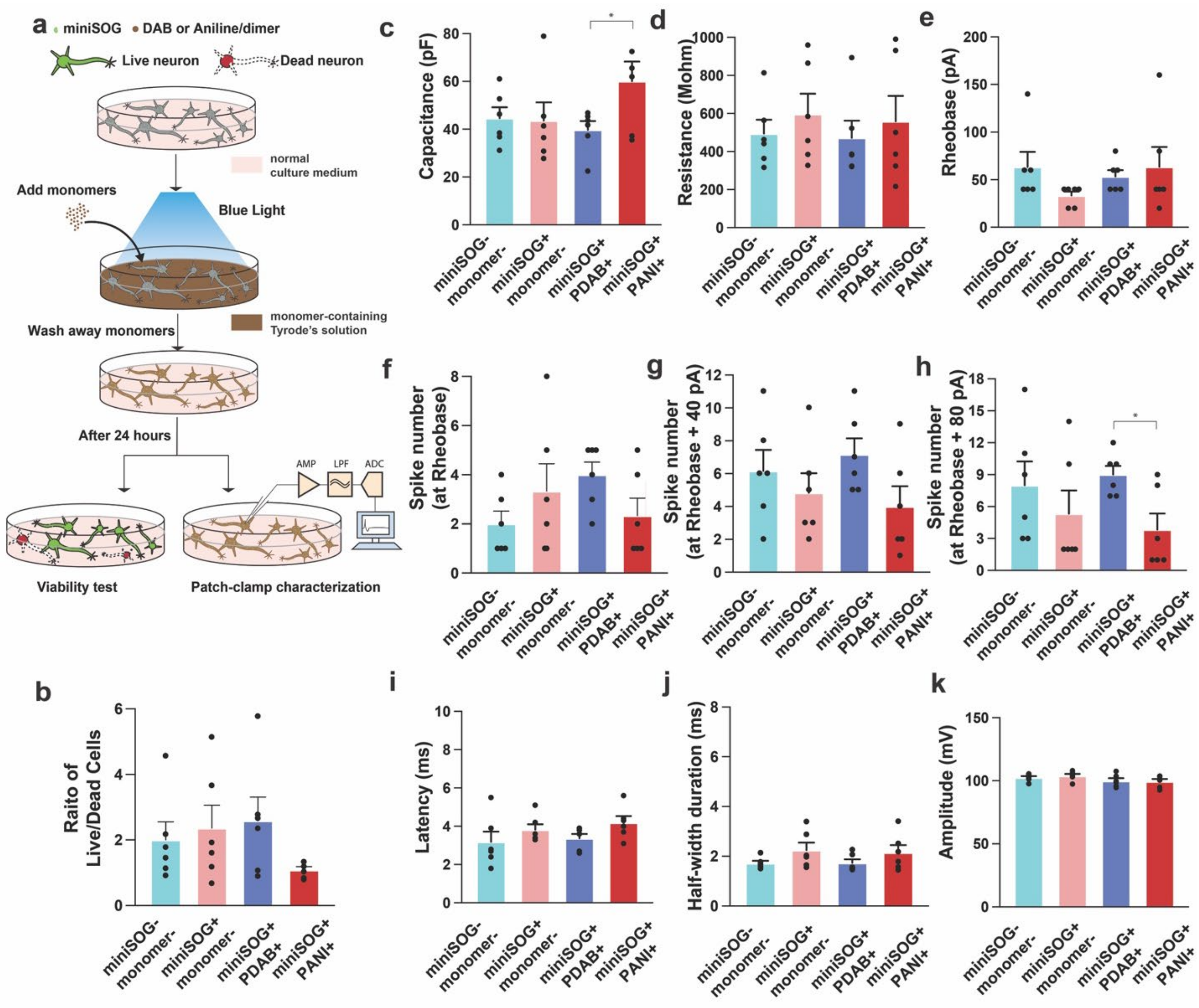

Supplementary Figure 13. Patch-clamp characterization and viability test 24 hours after optogenetic polymerization of PDAB and PANI. a, Schematic of the workflow for measurements of viability and electrophysiological characterization of cultured primary neurons 24 hours after optogenetic polymerization of PDAB and PANI. Neurons were perfused with either Tyrode's solution (monomer-), Tyrode's solution containing 0.5 mM aniline and 0.5 mM PPD (PANI+) or 1.0mM DAB (PDAB+) and irradiated with approximately 5 mW/mm^2 blue light that covers the whole plate. After irradiation, monomer or Tyrode's solutions were washed away and replaced with the original NbActiv4 medium and put back to the humid culture incubator with 5% CO_2 at 37 °C and incubated for another 24 hours. The neurons are then proceeded to viability test with the same configurations as in **Figure 2c** or whole-cell patch clamp characterizations. **b**, Statistical results of live/dead ratios. 6 groups per condition were performed. Values represent the ratio of live/dead cells. **c-e**, Membrane capacitance (**c**) and resistance (**d**) measured by 10 mV hyperpolarization step in voltage clamp, and the rheobase (**e**) determined by stepwise tonic current injection. **f-h**, Statistics of the stepwise tonic current-injection-evoked spikes at the rheobase (**f**), rheobase + 40 pA (**g**) and rheobase + 80 pA (**h**). i-k, Amplitude (**i**), latency (**j**) and half-width duration (**k**) extracted from spikes evoked by phasic currents (500 pA, 10 ms, 5 Hz) injection. n = 6 for each group. Bar graphs represent mean ± s.e.m. Unpaired two-tailed t-test. *$p < 0.05$.

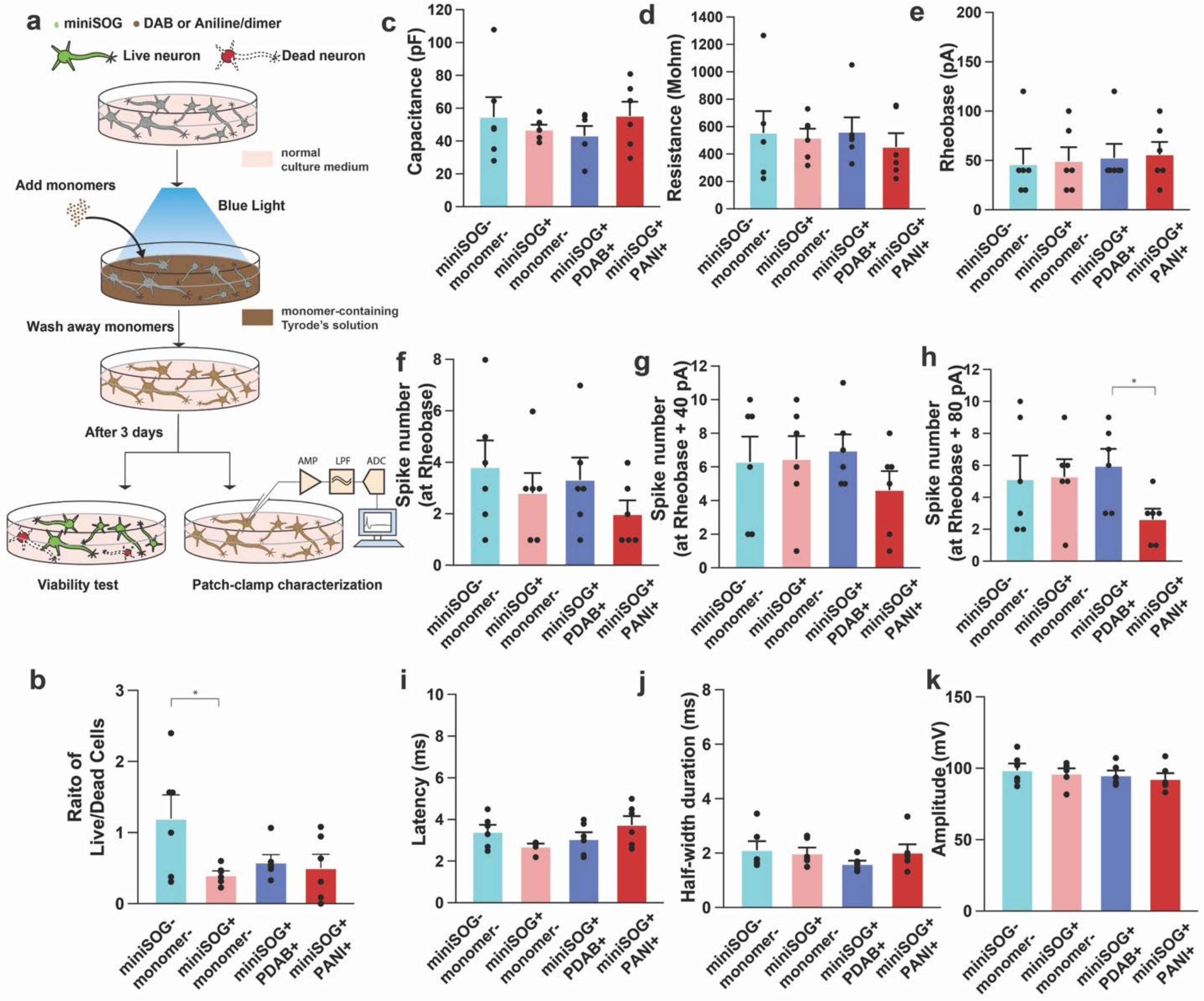

Supplementary Figure 14. Patch-clamp characterization and viability test 3 days after optogenetic polymerization of PDAB and PANI. a, Schematic of the workflow for measurements of viability and electrophysiological characterization of cultured primary neurons 3 days after optogenetic polymerization of PDAB and PANI with the same configurations as **fig. S13. b**, Statistical results of live/dead ratios. 6 groups per condition were performed. Values represent the ratio of live/dead cells. **c-e**, Membrane capacitance (**c**) and resistance (**d**) measured by 10 mV hyperpolarization step in voltage clamp, and the rheobase (**e**) determined by stepwise tonic current injection. **f-h**, Statistics of the stepwise tonic current-injection-evoked spikes at the rheobase (**f**), rheobase + 40 pA (**g**) and rheobase + 80 pA (**h**). **i-k**, Amplitude (**i**), latency (**j**) and half-width duration (**k**) extracted from spikes evoked by phasic currents (500 pA, 10 ms, 5 Hz) injection. n = 6 for each group. Bar graphs represent mean ± s.e.m. Unpaired two-tailed t-test. *$p < 0.05$.

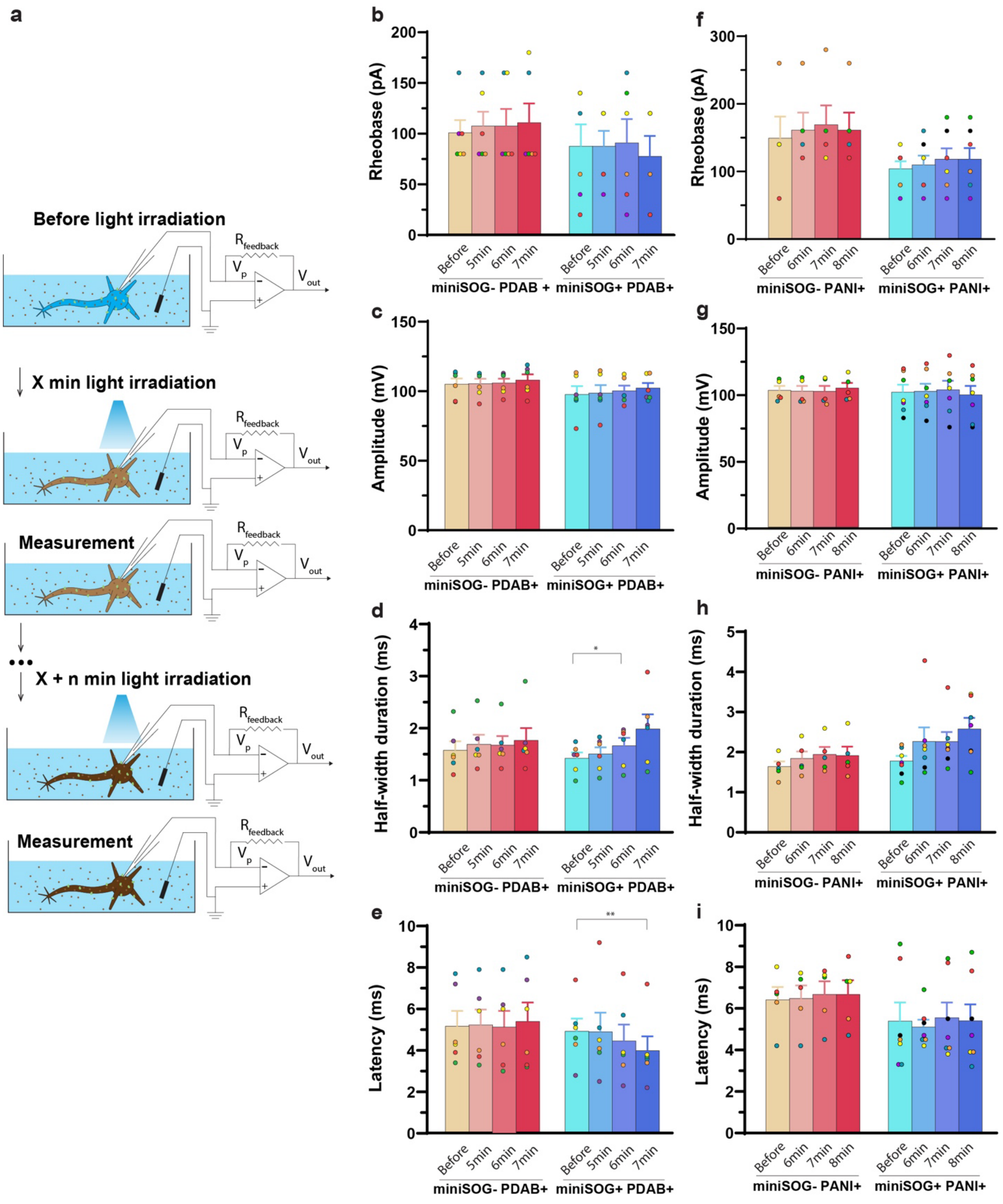

Supplementary Figure 15. Stepwise electrophysiological characterization of neurons before and after iterative optogenetic polymerization of PDAB and PANI. a, Schematics of electrophysiological recording on cultured neurons during the stepwise polymerization reaction. **b-e**, Change of rheobase (**b**), amplitude (**c**), half-width duration (**d**), and latency (**e**) before and after multiple rounds of blue light irradiation (~5 mW/mm^2, 5, 6, and 7 minutes) in the presence

of DAB monomers. **f-i**, Change of rheobase (**f**), amplitude (**g**), half-width duration (**h**), and latency (**i**) before and after multiple rounds of blue light irradiation in the presence of DAB monomers. All individual cells were maintained in the whole-cell configuration during the multiple rounds of irradiation for direct comparison. Bar graphs represent mean ± s.e.m, dots with the same color within each group indicate the same neuron. Paired two tailed t-test. *$p < 0.05$, **$p < 0.01$.

Synthesis of Biotin-DAB:

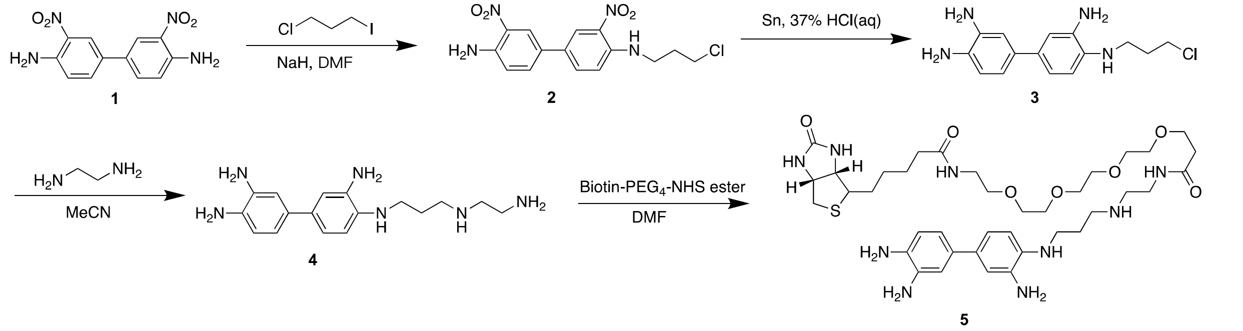

General synthesis information:

All chemicals/reagents were commercially available and used without further purification. The NMR spectra for both ^{1}H and ^{13}C were recorded from The Bruker Ascend 400 MHz NMR spectrometer at 298K with the deuterated solvent (DMSO-d_6) as the lock and the residual solvents as the internal reference.

Synthesis of dinitro benzidine-Cl (2): To a solution of 3,3'-dinitrobenzidine (1g, 3.646mmol) in dry DMF, (20ml) sodium hydride (131mg, 5.47mmol) was added in batches and the mixture was stirred at 0 °C for 30 mins. Then the mixture was added dropwise to a solution of 1-chloro-3-iodopropane (2.236g, 10.939mmol) in dry DMF (10ml). The mixture was then stirred under ambient condition and room temperature overnight. After the reaction was complete, the solvent was removed with rotary evaporator and then purified by column chromatography on silica gel using DCM/Hexanes (10% to 50% of DCM) as the eluent to afford compound 2 as a red solid (450mg, 35%). ^{1}H NMR (400 MHz, DMSO-d_6, 298 K) δ (ppm): 8.21 (d, J = 34.7 Hz, 3H), 7.83 (dd, J = 38.7, 8.8 Hz, 2H), 7.56 (s, 2H), 7.27 – 6.99 (m, 2H), 3.76 (s, 2H), 3.56 (s, 2H), 2.21 – 1.96 (m, 2H). ^{13}C NMR (101 MHz, DMSO-d6, 298 K) δ (ppm): 13C NMR (101 MHz, DMSO) δ 192.87, 151.34, 145.80, 144.46, 134.73, 134.09, 131.82, 130.84, 125.93, 122.81, 121.91, 120.60, 115.72, 43.50, 31.71.

Synthesis of DAB-Cl (3): compound **2** (150mg, 0.428mmol) and Sn powder (300mg, 2.53mmol) were dissolved in 5 ml of 37% hydrochloric acid and stirred at room temperature for 30mins. The mixture was then neutralized by saturated sodium bicarbonate solution, and then extracted with $CHCl_3$ for 3 x 20 ml each. The solvent was removed by rotary evaporator to afford compound **3** as light-yellow solid (100mg, 81%). ^{1}H NMR (400 MHz, DMSO-d_6, 298 K) δ (ppm): 6.89 – 6.74 (m, 2H), 6.64 (q, J = 14.2 Hz, 3H), 6.49 (dd, J = 18.5, 8.2 Hz, 1H), 5.20 (s, 7H), 3.79 (t, J = 6.5 Hz, 2H), 3.20 – 3.16 (m, 2H), 2.03 (h, J = 7.0 Hz, 2H).^{13}C NMR (101 MHz, DMSO-d_6, 298 K) δ (ppm): 135.29, 134.96, 132.78, 131.69, 130.61, 116.95, 116.28, 115.97, 113.66, 112.67, 110.88, 55.41, 43.95, 41.07, 32.18.

Synthesis of DAB-Amine (4): Compound **3** (50mg, 0.172 mmol) and ethylenediamine (1.0g, 16.65 mmol) were dissolved in 2ml of MeCN and the solution bubbled with nitrogen for 30 mins. The system was then heated to 60 °C and stirred under nitrogen atmosphere overnight. The solvent was then removed and the crude compounds purified by RP-HPLC (water/acetonitrile + 0.1% formic acid) to afford compound **4** as an orange solid (40mg, 74%).^{1}H NMR (400 MHz, DMSO-

d_6, 298 K, protonated) 8.39 (s, 3H), 6.73 (d, J = 2.1 Hz, 1H), 6.69 (d, J = 1.9 Hz, 1H), 6.64 (d, J = 8.0 Hz, 1H), 6.55 (d, J = 8.2 Hz, 1H), 6.49 (d, J = 7.9 Hz, 1H), 6.42 (d, J = 8.2 Hz, 1H), 6.36 (s, 8H), 3.09 (t, J = 6.8 Hz, 2H), 2.98 (s, 2H), 2.91 (d, J = 5.6 Hz, 2H), 2.81 (t, J = 7.1 Hz, 2H), 1.83 (p, J = 7.0 Hz, 2H).. ^{13}C NMR (101 MHz, DMSO-d6, 298 K) δ (ppm): 166.58, 135.80, 135.53, 134.81, 133.82, 131.25, 130.86, 115.30, 112.61, 112.26, 110.61, 46.53, 46.39, 41.77, 38.19, 37.72, 27.75.

Synthesis of Biotin-DAB (**5**): A 100 mM stock solution of compounds 4 in anhydrous DMSO was mixed with a 250 mM stock solution of Biotin-PEG$_4$-NHS ester in a 3:1 ratio (v/v) and stirred overnight at room temperature; the resulting solution was used for *in situ* polymerization without further purification. (ESI-MS: $[M+H]^+$: calculated m/z: 788.47, found m/z: 788.97)
